# MIG6 loss confers resistance to ALK/ROS1 inhibitors in NSCLC through EGFR activation by low-dose EGF

**DOI:** 10.1172/jci.insight.173688

**Published:** 2023-12-22

**Authors:** Nobuyuki Kondo, Takahiro Utsumi, Yuki Shimizu, Ai Takemoto, Tomoko Oh-hara, Ken Uchibori, Sophia Subat-Motoshi, Hironori Ninomiya, Kengo Takeuchi, Makoto Nishio, Yasunari Miyazaki, Ryohei Katayama

**Affiliations:** 1 Division of Experimental Chemotherapy, Cancer Chemotherapy Center, Japanese Foundation for Cancer Research (JFCR), Tokyo, Japan.; 2 Department of Respiratory Medicine, Tokyo Medical and Dental University, Tokyo, Japan.; 3 Department of Respiratory Medicine, Graduate School of Medical Sciences, Kyushu University, Fukuoka, Japan.; 4 Department of Computational Biology and Medical Science, Graduate School of Frontier Science, The University of Tokyo, Tokyo, Japan.; 5 Department of Thoracic Medical Oncology, the Cancer Institute Hospital,; 6 Department of Pathology, the Cancer Institute Hospital, and; 7 Pathology Project for Molecular Targets, Cancer Institute, JFCR, Tokyo, Japan.

**Keywords:** Oncology, Lung cancer

## Abstract

Although tyrosine kinase inhibitor (TKI) therapy shows marked clinical efficacy in patients with anaplastic lymphoma kinase–positive (ALK^+^) and ROS proto-oncogene 1–positive (ROS1^+^) non–small cell lung cancer (NSCLC), most of these patients eventually relapse with acquired resistance. Therefore, genome-wide CRISPR/Cas9 knockout screening was performed using an ALK^+^ NSCLC cell line established from pleural effusion without ALK-TKI treatment. After 9 days of ALK-TKI therapy, sequencing analysis was performed, which identified several tumor suppressor genes, such as NF2 or MED12, and multiple candidate genes. Among them, this study focused on ERRFI1, which is known as MIG6 and negatively regulates EGFR signaling. Interestingly, MIG6 loss induced resistance to ALK-TKIs by treatment with quite a low dose of EGF, which is equivalent to plasma concentration, through the upregulation of MAPK and PI3K/AKT/mTOR pathways. Combination therapy with ALK-TKIs and anti-EGFR antibodies could overcome the acquired resistance in both in vivo and in vitro models. In addition, this verified that MIG6 loss induces resistance to ROS1-TKIs in ROS1^+^ cell lines. This study found a potentially novel factor that plays a role in ALK and ROS1-TKI resistance by activating the EGFR pathway with low-dose ligands.

## Graphical abstract

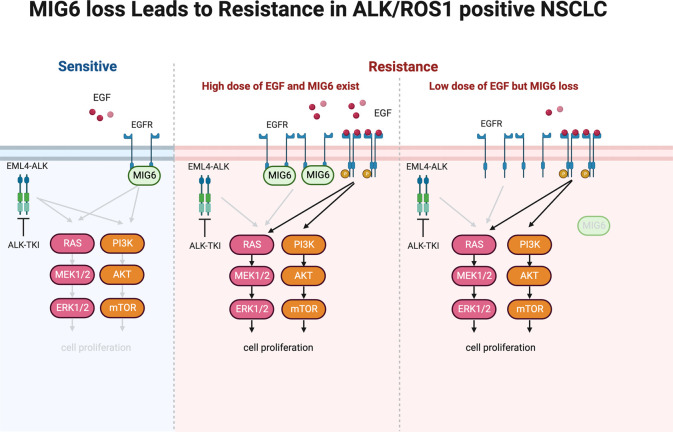

## Introduction

Lung cancer is the major cause of cancer-related deaths worldwide (1), and approximately 85% of lung cancers are non–small cell lung cancers (NSCLCs). Anaplastic lymphoma kinase (ALK) chromosomal rearrangement in NSCLC was originally described in 2007 (2) and affects approximately 3%–5% of patients with NSCLC (3–5). ALK fusion protein regulates several essential pathways involved in cell survival, proliferation, and cycling, including the PI3K/AKT/mTOR, RAS/MAPK, and JAK/STAT pathways (6, 7). Patients with NSCLC who have an ALK rearrangement respond remarkably to ALK tyrosine kinase inhibitors (ALK-TKIs). Thus far, the US FDA has approved 6 ALK-TKIs. Alectinib and brigatinib, the second-generation ALK-TKIs, and lorlatinib, a third-generation ALK-TKI, are widely used as standard treatments because of their high efficacy and manageable levels of toxicity (8, 9). Although ALK-TKIs significantly improve clinical outcomes, molecular target therapy will inevitably encounter acquired resistance. In approximately half of those receiving alectinib therapy, the disease will progress within 3 years (8).

The resistance mechanisms to ALK-TKIs are broadly classified into 2 categories: ALK-dependent and ALK-independent resistance. Mutations in the ALK kinase domain are the most common mechanisms of ALK-dependent resistance. ALK kinase domain mutations confer resistance to second-generation ALK-TKIs in approximately 50%–60% of cases (10). Following alectinib therapy, G1202R (occurring in 25%–30% of patients) and I1171X (occurring in 10%–15%) are the most frequent ALK-resistant mutations (10). Compound ALK mutations (such as C1156Y/L1198F, G1202R/L1196M, and I1171N/D1203N) account for the majority of on-target ALK mutations that confer resistance to lorlatinib (11, 12). Recently, we have reported that gilteritinib is effective against I1171N compound mutants (13). By contrast, examples of ALK-independent mechanisms include the activation of bypass signaling pathways, phenotypic modifications such as epithelial-mesenchymal transition (14) and small cell lung cancer (15), and drug efflux pump (16). The stimulation of bypass pathways by genetic changes or feedback signaling disruption is an important subset of an ALK-independent process. The hyperactivation of receptor tyrosine kinases has been reported to play an important role in resistance development. For example, the amplification of MET (17), HER2 (18), HER3 (19), and KIT (20) is associated with resistance, and high expression levels of IGF-1R ligands (21) mediate acquired resistance. Although the precise mechanism of EGFR activation is unclear, its signaling causes drug resistance (22). Activation of bypass pathways, such as receptor tyrosine kinase, has also been reported in the context of resistance mechanism to ALK-TKIs in anaplastic large cell lymphoma and neuroblastoma (23). In addition, reactivations of downstream effector proteins such as MAP2K1 (24), DUSP6 (25), and STAT3 (26) have been recognized as bypass pathways. As a resistance mechanism, the functional deletion of tumor suppressor genes, including NF2 (14) and MED12 (27), was also discovered. Combination strategies may be used to overcome resistance because ALK-TKI–resistant cells frequently still have a partial reliance on ALK for proliferation (28). Although numerous sophisticated studies have contributed to the better understanding of off-target resistance to ALK-TKIs, a sizable portion of resistance mechanisms remain unclear. Therefore, more studies are needed to elucidate the underlying resistance to ALK-TKIs to improve clinical outcomes in patients with ALK-positive NSCLC.

ROS proto-oncogene 1 (ROS1) gene rearrangements are observed in 1% of patients with NSCLC (29–31). Tumor growth is facilitated by ROS1 fusion protein–induced constitutive activation of ROS1 tyrosine kinase. Many ALK-TKIs can also successfully bind to ROS1 kinase because the kinase domains of ROS1 and ALK have a substantial amount of homology. ROS1-TKIs crizotinib and entrectinib have been approved in several countries and show considerable improvement in patients with ROS1-positive NSCLC (32, 33); however, complete remission is uncommon. ROS1 G2032R is the most common resistance substitution, which is analogous to ALK G1202R (34). Resistance to ROS1-TKIs has also been linked to the activation of bypass or downstream mediators, such as EGFR, HER2, MET, KRAS, BRAF, and KIT (35–39). However, the resistance mechanism remains unknown in a substantial proportion of patients.

Emerging evidence shows that small subpopulations of cancer cells called drug tolerant persister (DTP) cells can survive under molecular target therapy and play a critical role in disease progression by mediating drug resistance (40). DTP cells are characterized by a reversible slow proliferation state that is controlled by metabolic remodeling, interactions with the tumor microenvironment, transcriptional processes, and genetic or epigenetic modifications (41–45). DTP state can allow cancer cells to escape from target therapy and serve as a reservoir for the development of diverse drug resistance mechanisms upon long-term therapy (46). Thus, to develop therapeutic approaches for DTP, more studies are needed for a better understanding of drug resistance.

CRISPR/Cas9 is a simple and accurate gene-engineering tool using a 20 bp single-guide RNA (sgRNA) and a Cas9 protein, which can specifically detect and cut the sgRNA-binding region (47). Recently, genome-wide CRISPR/Cas9 pooled screening allow us to uncover drug resistance mechanisms in multiple cancer types, including NSCLC and melanoma (48–50). In this study, we performed a genome-wide knockout CRISPR/Cas9 library screening in an ALK-positive NSCLC line derived from pleural effusion without ALK-TKI therapy. Following the analysis of sgRNA sequencing, we discovered *ERRFI1*, known as MIG6, which binds EGFR and negatively regulates it, as being responsible for the resistance to ALK-TKIs. Combination therapy with anti-EGFR antibody resensitized MIG6-knockout cells to ALK inhibition. Similar results were obtained in ROS1-positive cell lines. Our data suggest that MIG6 is a potential therapeutic target to overcome the resistance mechanism to ALK- and ROS1-TKIs.

## Results

### MIG6 was identified as a gene responsible for ALK inhibitor resistance using CRISPR library screening.

**Figure 1:**
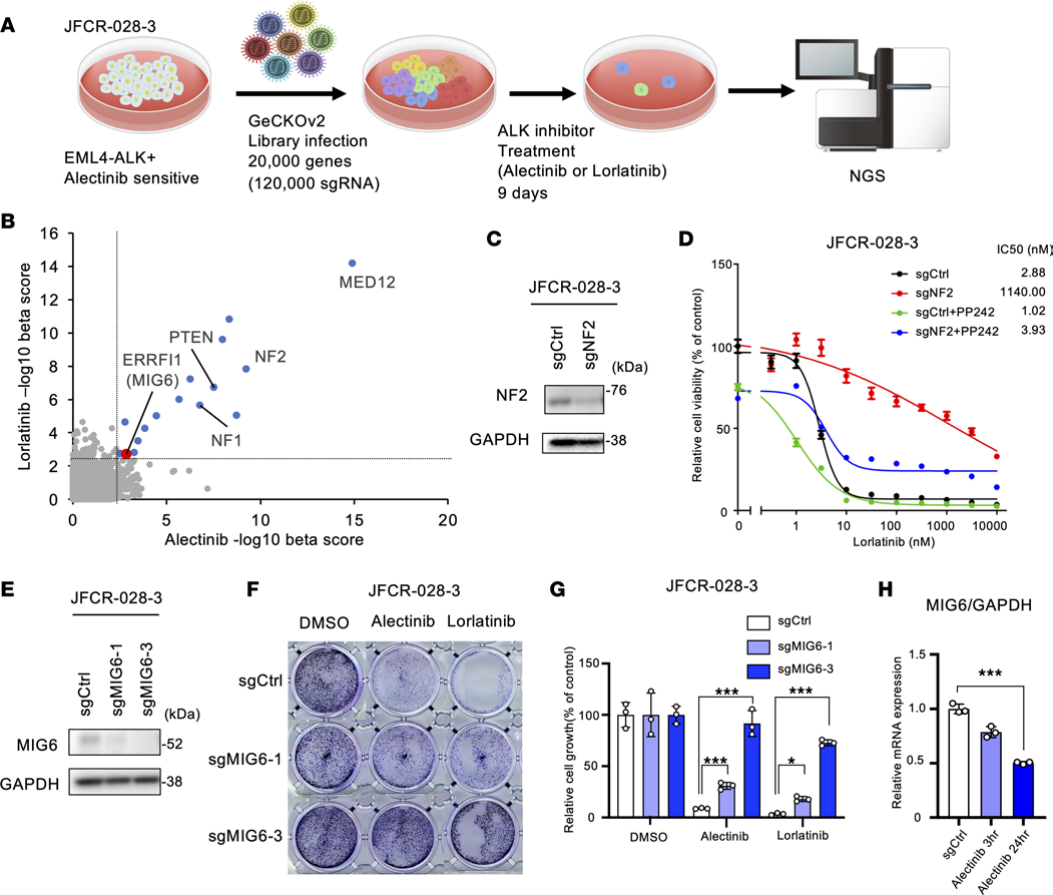
CRISPR library screening identifies MIG6 depletion in ALK-TKI–resistant cells. (**A**) Schematic diagram of the workflow of genome-wide CRISPR/Cas9 library screening to identify critical genes related to alectinib and lorlatinib resistance in the JFCR-028-3 cell line. NGS, next-generation sequencing. (**B**) The abundance of sgRNA for each gene in CRISPR library screening was evaluated by the β-score using the MAGeCK algorithm. Positively selected genes after both alectinib and lorlatinib treatments (cutoff of –log_10_ β-score > 2.5) are indicated as blue dots. MIG6 (ERRFI1) is indicated as a red dot. (**C**) Immunoblot analysis of NF2 knocked out in JFCR-028-3 cells. (**D**) JFCR-028-3 cells were treated with the indicated concentrations of lorlatinib with or without 1 μmol/L of PP242 for 72 hours. Cell viability was measured using the CellTiter-Glo assay (*n* = 3). (**E**) Immunoblot analysis of MIG6 knocked out in JFCR-028-3 cells. (**F** and **G**) Colony formation assays were performed in JFCR-028-3 cells. JFCR-028-3 sg-control (Cntl) or sg-MIG6 cells were treated with 10 nmol/L of alectinib or 3 nmol/L of lorlatinib for 2 weeks. Surviving cells were stained with crystal violet. Representative images are shown in **F**. Relative cell viability was measured using a spectrophotometer after solubilizing the stained crystal violet with the acetic acid buffer from each well (**G**). (**H**) Quantitative reverse transcription PCR (RT-qPCR) of MIG6 mRNA was performed using JFCR-028-3 cells treated with 300 nmol/L of alectinib for the indicated hours. (**C**–**H**) Similar experiments were performed twice (**C** and **E**) or 3 times (**D** and **F**–**H**), and representative data are shown. Each point represents mean ± SD of 3 technical replicates; *P < 0.05, ***P < 0.001 (2-way ANOVA following Dunnett post hoc test).

To identify genes whose loss of function confers ALK-TKI tolerance or resistance, a genome-wide knockout CRISPR/Cas9 screening was performed (Figure 1A) in a patient-derived JFCR-028-3 cell line, which was obtained from pleural effusion. As previously reported (51), JFCR-028-3 cells are susceptible to ALK-TKIs but resistant to EGFR-TKIs (Supplemental Figure 1A; supplemental material available online with this article; https://doi.org/10.1172/jci.insight.173688DS1). First, JFCR-028-3 cell lines were engineered to overexpress Cas9 using lentivectors and single-cell cloning was performed. Knockout efficacy was evaluated using sgRNA targeting EpCAM (Supplemental Figure 1B). Sensitivity to ALK-TKIs in Cas9-overexpressed cells was compatible to that of the parental cells (Supplemental Figure 1C). Then, JFCR-028-3 cells were transfected with human genome-scale CRISPR-knockout libraries A and B (52). Transduced cells were treated with 300 nmol/L of alectinib, 100 nmol/L of lorlatinib, or DMSO for 9 days. Genomic DNA was collected and next-generation sequencing was performed. The abundance of sgRNA for each gene was evaluated by computing the β-score using the MAGeCK algorithm (53) by comparing ALK-TKI with DMSO treatment. A total of 14 genes, including *MED12*, *NF1*, *NF2*, and *PTEN*, were shown to be enriched after both alectinib and lorlatinib therapy (Figure 1B). In the past *NF2* has been described as a tumor suppressor gene by inhibiting PI3K/AKT/mTOR signaling (14). *NF2* knockouts were created in JFCR-028-3 cells to validate CRISPR screening (Figure 1C). NF2-knockout cells showed decreased sensitivity to ALK-TKIs (Figure 1D and Supplemental Figure 2). ALK-TKI sensitivity was restored by the addition of the mTOR inhibitor PP242 (Figure 1D and Supplemental Figure 2). These results suggest that CRISPR screening was conducted successfully.

Then, we focused on MIG6, known as the EGFR feedback protein, because EGFR signaling was reported to be essential for the ALK-TKI resistance mechanism (22). MIG6 was depleted in JFCR-028-3 cells using 2 independent sgRNAs, and knockout efficacy was verified (Figure 1E). MIG6 depletion resulted in increased number of DTP cells in a long-term treatment model (Figure 1, F and G, and Supplemental Figure 3). Since sgMIG6-3 showed high knockout efficacy, we focused on sgMIG6-3 for detection of resistance mechanisms in more detail. Similar results were observed in H3122 cells, an ALK-positive NSCLC cell line (Supplemental Figure 4). We also evaluated whether ALK inhibition is related to suppression of MIG6. Following ALK-TKI therapy, expression levels of MIG6 were decreased, whereas those of EGF and TGF-α were increased (Figure 1H and Supplemental Figure 5). These findings indicated that MIG6 depletion is related to resistance to ALK-TKIs.

### Low-dose EGFR ligands confer more resistance to ALK inhibitors in MIG6-knockout cells.

**Figure 2:**
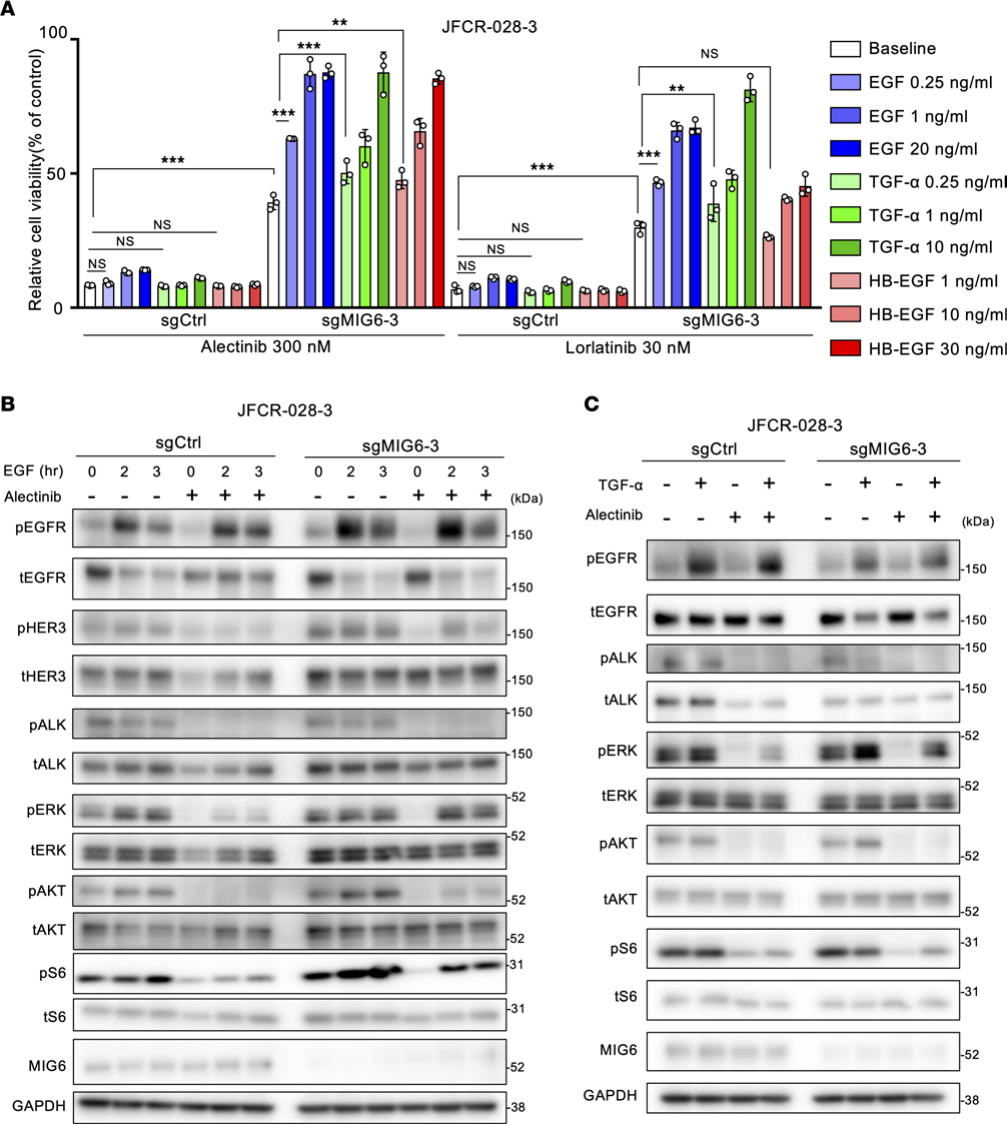
Low-dose EGFR ligands confer more resistance to ALK inhibitors in MIG6-knockout cells. (**A**) JFCR-028-3 cells were treated with the indicated concentrations of drugs and ligands for 72 hours. Cell viability was measured using the CellTiter-Glo assay. Each point represents the mean ± SD of 3 technical replicates; **P < 0.01, ***P < 0.001 (2-way ANOVA following Tukey’s post hoc test). (**B** and **C**) Protein expression of the downstream pathway of ALK in JFCR-028-3 cells. Cells were treated with 300 nmol/L of alectinib for 3 hours and 20 ng/mL of EGF for the indicated hours (**B**) or 10 ng/mL of TGF-α for 3 hours (**C**). (**A**–**C**) Similar experiments were performed twice (**B** and **C**) or 3 times (**A**), and representative data are shown.

MIG6-knockout cells showed more resistance to ALK-TKIs than control cells (Figure 2A). Since MIG6 is a feedback protein of ErbB receptors, whether EGF ligands affect the sensitivity of ALK inhibitors was examined. As previously reported (54), high doses of EGF, TGF-α, and HB-EGF induced resistance even in control cells. On the contrary, low doses of EGFR ligands conferred resistance only in MIG6-knockout cells (Figure 2A and Supplemental Figure 6A). Previous studies have reported that these concentrations of EGFR ligands at “low doses” are equivalent to serum concentrations in patients with NSCLC and colorectal cancer (55, 56). In MIG6-knockout cells, baseline phosphorylation levels of MAPK and PI3K/AKT/mTOR pathways were higher (Figure 2B). Consistent with the cell viability assay result, downstream ALK pathways were upregulated in MIG6-knockout cells by ALK-TKI therapy with EGFR ligands (Figure 2, B and C). Similar results were observed in H3122 cells (Supplemental Figure 6B and Supplemental Figure 7). Interestingly, the protein expression levels in MIG6-depleted cells exhibited increased activation of HER3 (Figure 2B). These results indicate that the proliferation of MIG6-knockout cells is highly susceptible to EGFR ligands.

### Combination therapy with EGFR inhibitors and ALK-TKI could overcome the resistance related to MIG6 depletion.

**Figure 3:**
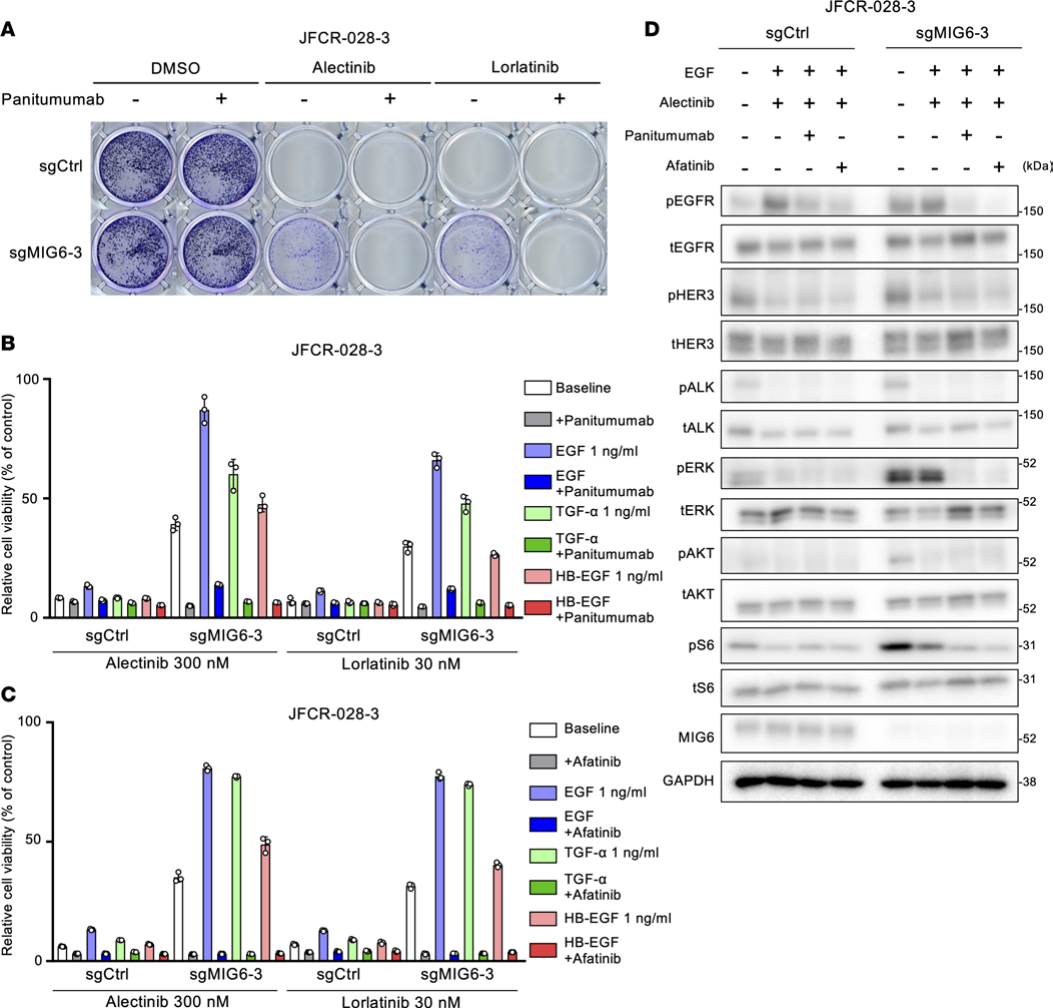
Combination therapy with EGFR inhibitors and ALK-TKIs can overcome the resistance related to MIG6 depletion. (**A**) Colony formation assays were performed in JFCR-028-3 cells. Each well was treated with 100 nmol/L of alectinib or 30 nmol/L of lorlatinib with or without 10 μg/mL of panitumumab for 2 weeks, and surviving cells were stained with crystal violet. Representative images are shown. (**B** and **C**) JFCR-028-3 cells were treated with the indicated concentrations of ALK-TKIs and ligands with or without 10 μg/mL of panitumumab (**B**) or 100 nmol/L of afatinib (**C**) for 72 hours. Cell viability was measured using the CellTiter-Glo assay. Each point represents the mean ± SD of 3 technical replicates. (**D**) Protein expression of the downstream pathway of ALK in JFCR-028-3 cells. Cells were treated with 300 nmol/L of alectinib, 10 μg/mL of panitumumab, 100 nmol/L of afatinib, and 1 ng/mL of EGF for 3 hours. (**A**–**D**) Similar experiments were performed twice (**D**) or 3 times (**A**–**C**), and representative data are shown.

We hypothesized that by preventing EGFR ligands from binding to EGFR, MIG6-knockout cells could regain their sensitivity to ALK-TKIs. To verify this, colony formation and cell viability assays were performed using combination therapy with ALK-TKIs and panitumumab, an anti-EGFR antibody. The resistance related to MIG6 knockout could be successfully overcome by combination therapy with panitumumab (Figure 3, A and B, and Supplemental Figure 8, A and B). Tumor cell proliferation could also be suppressed by the combination therapy with ALK-TKIs and afatinib, a pan-ErbB inhibitor (Figure 3C). Notably, MIG6-knockout cells were resistant to EGFR-TKIs (Supplemental Figure 9), suggesting that the proliferation of these cells mainly depends on the ALK pathway. Consistently, downregulation of both EGFR and ALK suppressed the downstream pathways of ALK in MIG6-knockout cells (Figure 3D, Supplemental Figure 6C, and Supplemental Figure 9). These results indicate that the inhibition of both ALK and EGFR could be a potential target to overcome MIG6 depletion–related resistance.

### MIG6 loss conferred resistance to ALK inhibitors in vivo.

**Figure 4:**
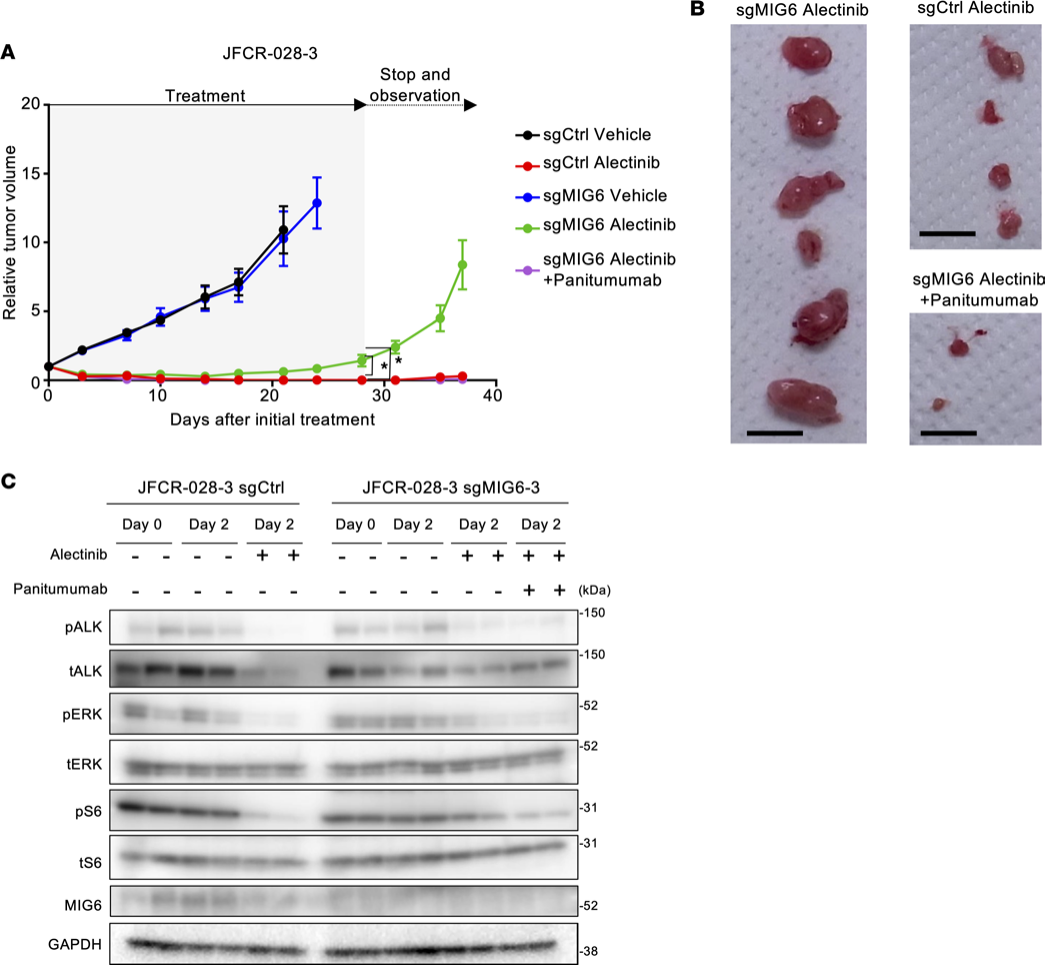
MIG6 depletion confers resistance to ALK inhibitors in vivo. (**A** and **B**) JFCR-028-3 control and MIG6-knockout cells were subcutaneously injected into BALB/c nude mice. The mice were treated with vehicle, alectinib (10 mg/kg) orally, or alectinib plus panitumumab (0.5 mg, twice a week) intraperitoneally for 4 weeks (*n* = 6). Data are presented as the mean ± SEM; *P < 0.05 (1-way ANOVA following Dunnett’s test). Images of xenograft tumors on day 37 after the initial treatment are shown in **B**. The black bar indicates 1 cm. (**C**) JFCR-028-3 sg-control or sgMIG6 tumor–bearing mice were treated with alectinib, with or without panitumumab for 2 days, and 3 hours after the treatment on day 2, mice were euthanized; the tumors were taken for immunoblot analysis with the indicated antibodies.

Then, the antitumor effect of alectinib plus panitumumab was evaluated in patient-derived cell (PDC) models in vivo (Figure 4A and Supplemental Figure 10A). In JFCR-028-3 control xenografts treated with alectinib monotherapy and MIG6-knockout JFCR-028-3 xenografts treated with both alectinib and panitumumab, tumors significantly shrank. While alectinib monotherapy demonstrated tumor regression in MIG6-knockout cells, small tumors that were present during alectinib therapy regrew after drug therapy discontinuation. Combination therapy with alectinib and panitumumab resulted in tumor disappearance, without weight loss (Supplemental Figure 10B and Supplemental Figure 11). Surprisingly, these tumors showed little regrowth even after treatment cessation (Figure 4B). Consistently, the downstream pathways of ALK remained activated in MIG6-knockout xenografts treated with alectinib monotherapy, and the combination therapy with alectinib and panitumumab suppressed the activation of those (Figure 4C). Notably, MIG6-knockout xenografts were resistant to panitumumab monotherapy (Supplemental Figure 12). These findings suggest that MIG6 depletion conferred resistance to ALK-TKIs and that combination therapy with ALK-TKIs and panitumumab was similarly effective in vivo.

### Decreased MIG6 expression levels in clinical samples.

**Figure 5:**
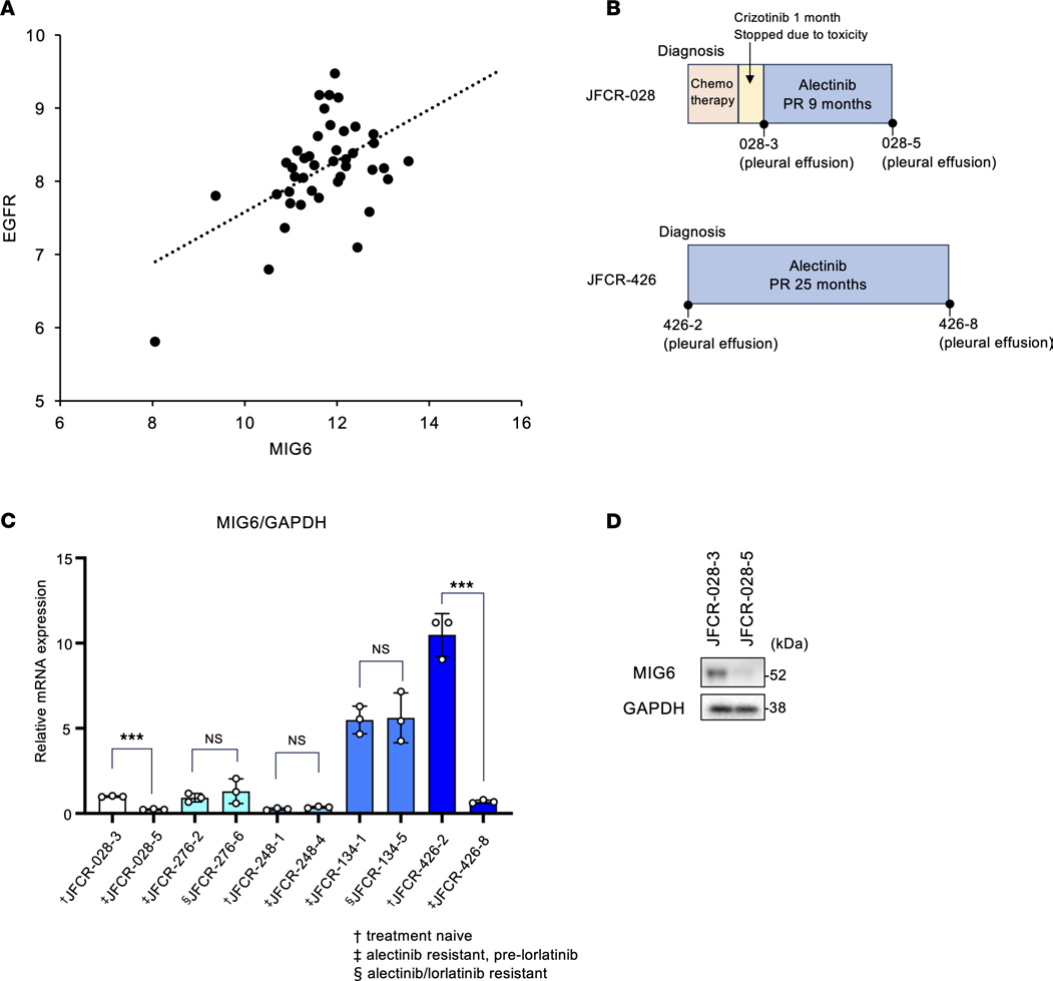
Some ALK-TKI–resistant clinical samples show decreased MIG6 levels. (**A**) Microarray data were analyzed from 42 patients with ALK-positive lung cancer who underwent lung surgery at our hospital (GSE128309). Relative expression levels of MIG6 and EGFR (log_2_) are shown. (**B**) Clinical course of patient JFCR-028 and JFCR-426. PR, partial response. (**C**) RT-qPCR of ERRFI1 was performed using clinical samples. Each point represents the relative mRNA expression of MIG6/GAPDH shown as mean ± SD of 3 technical replicates; ***P < 0.001 (Student’s *t* test). (**D**) Immunoblot analysis of MIG6 in JFCR-028-3 or 028-5 cells. Similar experiments were performed twice.

To evaluate the correlation between MIG6 expression and clinical outcomes, this study analyzed microarray data from 42 patients with ALK-positive lung cancer who underwent lung surgery at our hospital (National Center for Biotechnology Information Gene Expression Omnibus GSE128309). Consistent with in vitro data, relative MIG6 expression level revealed a broad variation from 1- to 44-fold and was moderately correlated with EGFR expression (Figure 5A). Since few patients received ALK-TKI therapy, the relationship between MIG6 expression level and clinical outcomes could not be determined. Therefore, 5 ALK-positive patients who had relapsed on alectinib or lorlatinib were examined. A total of 3 of 5 cases demonstrated approximately consistent mRNA expression levels of MIG6 between ALK-TKI–sensitive and –resistant specimens. JFCR-028 and JFCR-426 showed decreased MIG6 expression levels in the alectinib-resistant samples (Figure 5, B and C, and Supplemental Table 1). Moreover, compared with alectinib-sensitive JFCR-028-3, alectinib-resistant JFCR-028-5 displayed lower levels of MIG6 protein expression (Figure 5D). As previously described, JFCR-028-5 cells acquired resistance to ALK-TKIs by activation of Src and EGFR (51). The decreased MIG6 expression levels in JFCR-028-5 cells were consistent with the EGFR signaling activation. These data suggest that decreased MIG6 expression might be correlated to ALK-TKI resistance in clinical samples.

### MIG6 depletion conferred resistance to ROS1-TKIs in ROS1-rearranged NSCLC cell lines.

**Figure 6:**
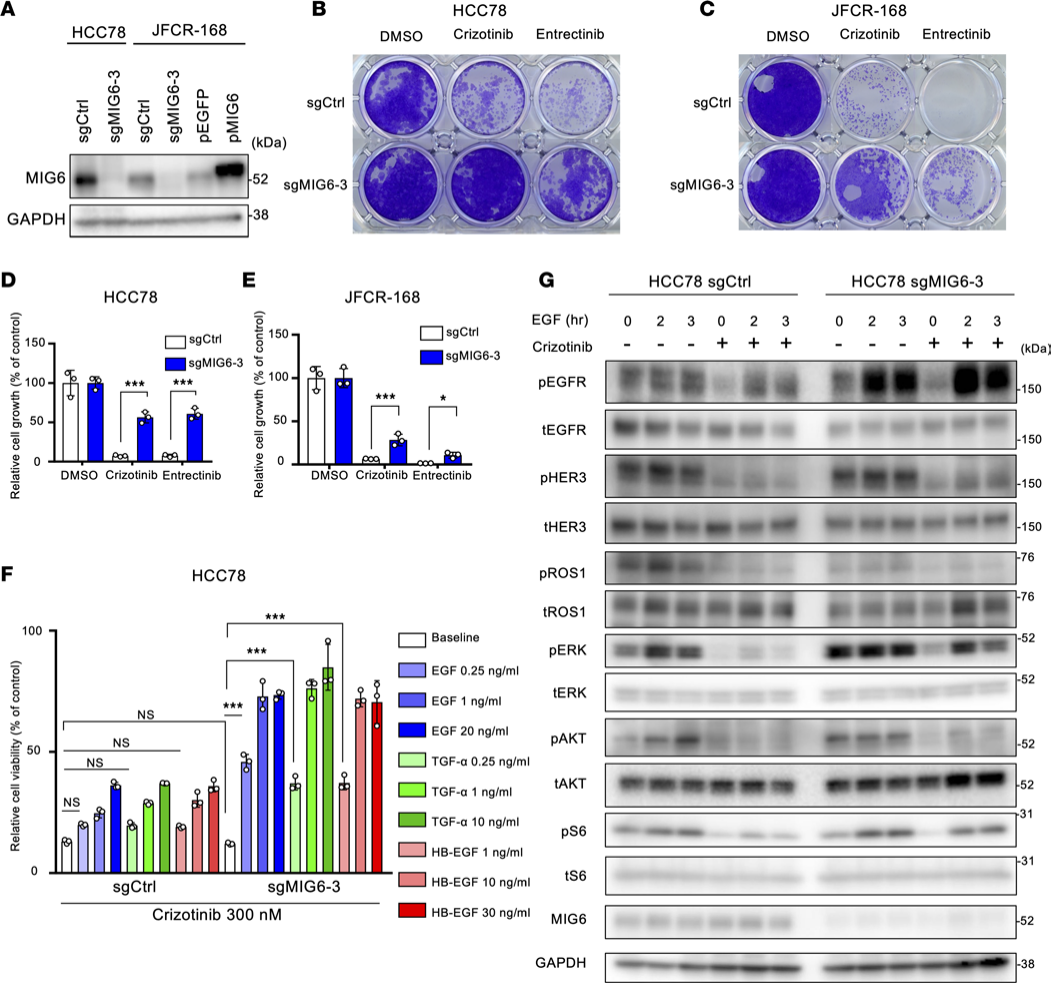
MIG6 depletion confers resistance to ROS1-TKIs in ROS1-rearranged NSCLC cell lines. (**A**) Immunoblot analysis of MIG6 knocked out in HCC78 cells and JFCR-168 cells. JFCR-168 cells were also induced to overexpress MIG6. (**B**–**E**) Colony formation assays were performed in HCC78 (**B** and **D**) and JFCR-168 (**C** and **E**) cells. Each well was treated with 1,000 nmol/L of crizotinib or entrectinib for 9 days to 2 weeks, and surviving cells were stained with crystal violet. Representative images are shown in **B** and **C**. Relative cell viability was measured using a spectrophotometer after solubilizing the stained crystal violet with an acetic acid buffer from each well (**D** and **E**). (**F**) HCC78 cells were treated with the indicated concentrations of crizotinib and ligands for 72 hours. Cell viability was measured using the CellTiter-Glo assay. (**G**) Protein expression of the downstream pathway of ROS1 in HCC78 cells. Cells were treated with 1,000 nmol/L of crizotinib for 3 hours and 20 ng/mL of EGF for the indicated hours. (**A**–**G**) The results indicate the mean ± SD of 3 technical replicates; *P < 0.05, ***P < 0.001 (2-way ANOVA following Tukey’s post hoc test). Similar experiments were performed twice (**A** and **G**) or 3 times (**B**–**F**), and representative data are shown.

Owing to the high amino acid similarities in the kinase domains of ALK and ROS1, several ALK-TKIs, such as crizotinib or lorlatinib, have antitumor effects on ROS1-rearranged cell lines (57) and showed clinical benefits in patients with ROS1-positive NSCLC (58, 59). Therefore, we hypothesized that the resistance mechanism of MIG6 depletion could be applicable in ROS1-rearranged cancers. To confirm this, MIG6-knockout cell line was established in HCC78, which harbors *SLC34A2-ROS1* rearrangement, and in JFCR-168, which was obtained from the pleural effusion and expresses the *CD74-ROS1* fusion gene (Figure 6A). MIG6 depletion in ROS1-rearranged cell lines showed resistance to ROS1-TKIs in long-term treatment models (Figure 6, B–E, and Supplemental Figure 13).

Subsequently, the effect of EGFR ligands on cell viability was examined. As expected, low-dose (no more than 1 ng/mL) EGFR ligands conferred resistance to ROS1-TKIs in MIG6-knockout HCC78 cells (Figure 6F and Supplemental Figure 14A). On the other hand, only high doses of EGFR ligands caused resistance in control cells (Figure 6F and Supplemental Figure 14A). Similar tendencies were observed in JFCR-168 cells (Supplemental Figure 14B). Consistent with the cell viability assay result, downstream pathways of ROS1 were upregulated in MIG6-knockout HCC78 cells treated with EGFR ligands and ROS1-TKIs (Figure 6G and Supplemental Figure 14C).

Because the MIG6 expression level in JFCR-168 control cells was much lower than that in HCC78 control cells (Figure 6A), we hypothesized that quite a low dose of EGF could lead to ROS1-TKI resistance in these cells. Indeed, the proliferation of control cells increased at relatively low doses of EGF (Supplemental Figure 15). However, consistent with the results of the colony formation assays, levels of apoptotic markers decreased in MIG6-knockout cells treated with both EGF and ROS1-TKIs (Supplemental Figure 14D). Then, this study investigated whether ROS1-TKI sensitivity could be restored by high MIG6 expression levels. To determine this, MIG6-overexpressed cells were established in JFCR-168 (Figure 6A). As expected, MIG6 overexpression inhibited cell viability induced by EGFR ligands with ROS1 inhibitors (Supplemental Figure 15). High expression levels of MIG6 suppressed the EGF-induced upregulation of MAPK and PI3K/AKT/mTOR pathways (Supplemental Figure 16). Together, these results support that MIG6 depletion also led to the development of resistance to ROS1-TKIs.

### Combination therapy with EGFR inhibitors and ROS1-TKIs could overcome MIG6 depletion–related resistance.

**Figure 7:**
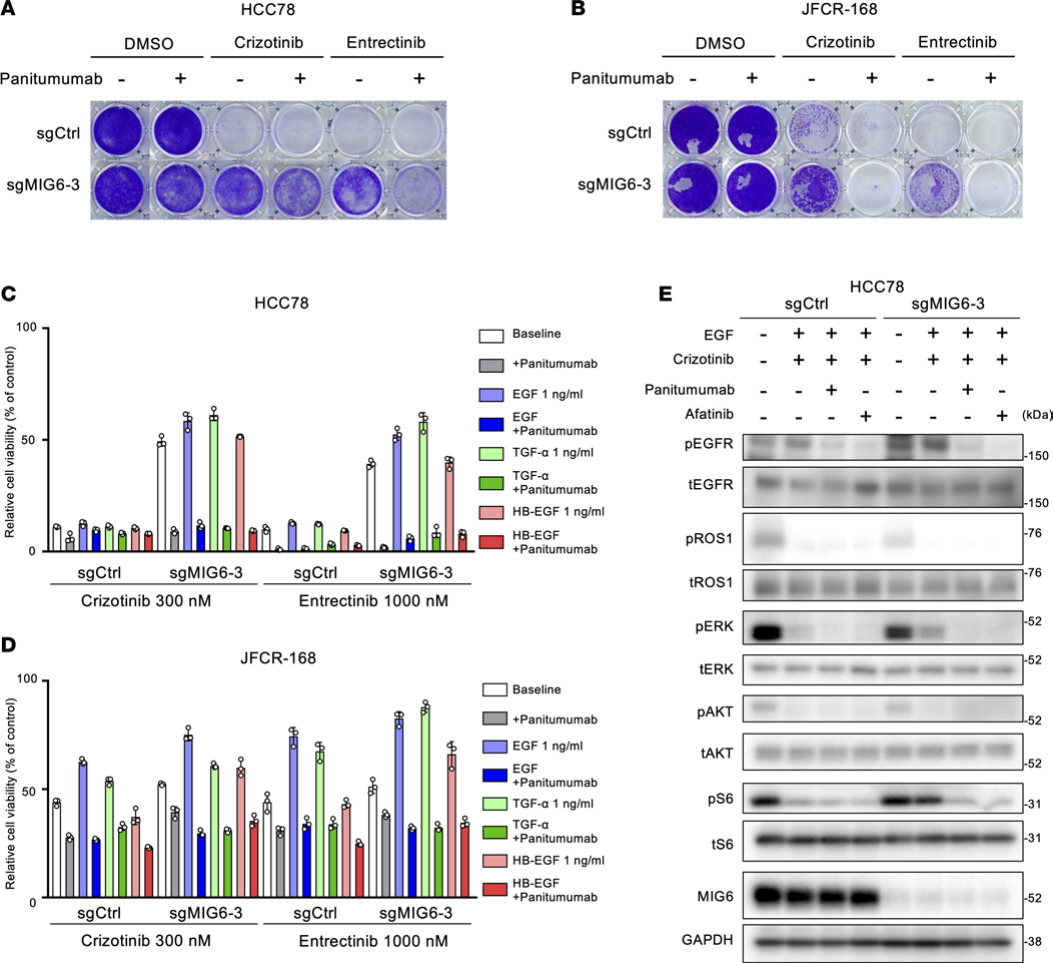
Resistance resulting from MIG6 depletion can be overcome by combining EGFR inhibitors and ROS1-TKIs. (**A** and **B**) Colony formation assays were performed in HCC78 (**A**) and JFCR-168 (**B**) cells using 3 technical replicates. Each well was treated with 1,000 nmol/L of crizotinib or entrectinib with or without 10 μg/mL of panitumumab for 9 to 14 days, and surviving cells were stained with crystal violet. Representative images are shown. (**C** and **D**) HCC78 (**C**) and JFCR-168 (**D**) cells were treated with the indicated concentrations of ROS1-TKIs and ligands with or without 10 μg/mL of panitumumab for 72 hours. Cell viability was measured using the CellTiter-Glo assay. Each point represents the mean ± SD of 3 replicates. (**E**) Protein expression of the downstream pathway of ROS1 in HCC78 cells. Cells were treated with 1,000 nmol/L of crizotinib, 10 μg/mL of panitumumab, 100 nmol/L of afatinib, and 1 ng/mL of EGF for 3 hours. (**A**–**E**) Similar experiments were performed twice (**B**–**E**) or 3 times (**A**), and representative data are shown.

The antitumor efficacy of combination therapy with crizotinib and panitumumab was evaluated in MIG6-knockout cells. Combination therapy with panitumumab could successfully restore the sensitivity to crizotinib in MIG6-knockout cells in long-term treatment models (Figure 7, A and B). Even after treatment with EGFR ligands, combination therapy with panitumumab could nearly entirely resensitize MIG6-knockout cells to ROS1-TKIs (Figure 7, C and D, and Supplemental Figure 17A). Consistently, the inhibition of both EGFR and ROS1 suppressed the downstream pathways of ROS1 in MIG6-knockout cells (Figure 7E and Supplemental Figure 17B). Combination therapy with panitumumab induced increased levels of apoptosis in MIG6-knockout JFCR-168 cells (Supplemental Figure 17C). These results indicate that the inhibition of both ROS1 and EGFR could also be a potential target to overcome MIG6 depletion–related resistance.

### Panitumumab with ROS1-TKIs prevents MIG6-knockout tumor regrowth in vivo.

**Figure 8:**
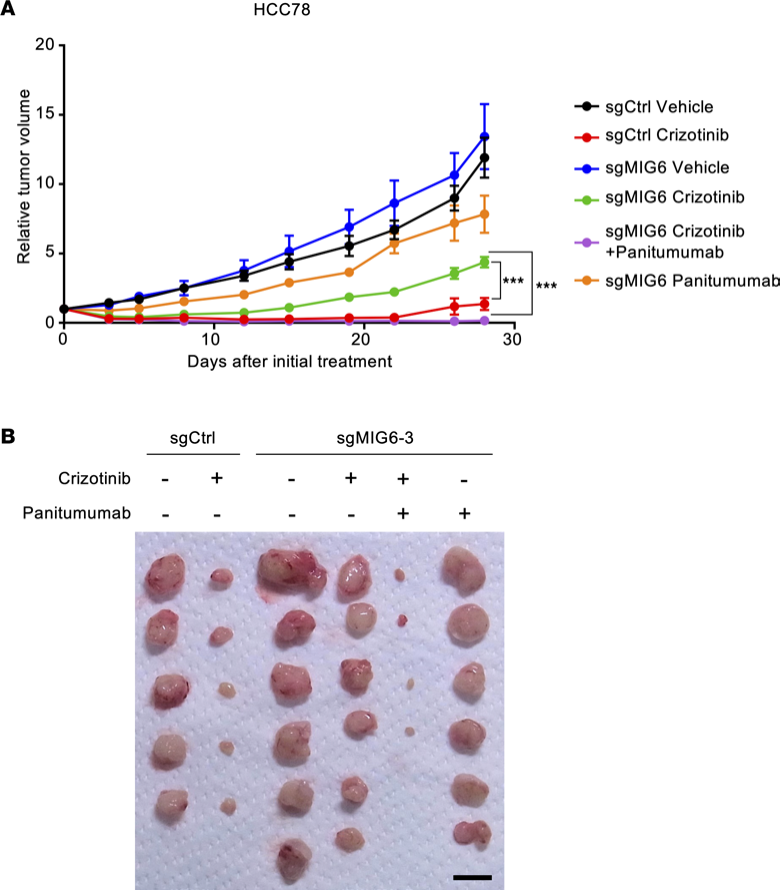
Antitumor effect of ROS1-TKIs in the HCC78 xenograft MIG6 depletion model. (**A** and **B**) HCC78 control and MIG6-knockout cells were subcutaneously transplanted into BALB/c nude mice. The mice were treated with vehicle, crizotinib (50 mg/kg) orally, panitumumab (0.5 mg, twice a week) intraperitoneally, or crizotinib plus panitumumab for 4 weeks (*n* = 5–6). Data are presented as the mean ± SEM; ***P < 0.001 (1-way ANOVA following Dunnett’s test). Images of xenograft tumors on day 28 after the initial treatment are shown in **B**. The black bar indicates 1 cm.

Then, the antitumor effect on MIG6-depleted cells was evaluated using an in vivo xenograft mouse model as previously described (60). HCC78 control and MIG6-knockout cells were subcutaneously implanted into nude mice and treated with crizotinib alone, panitumumab alone, or crizotinib and panitumumab. Combination therapy with crizotinib and panitumumab induced rapid tumor regression in HCC78 MIG6-knockout xenografts (Figure 8A and Supplemental Figure 18A). On the contrary, crizotinib monotherapy induced significant tumor shrinkage only in the control xenograft. While treatment with crizotinib monotherapy induced tumor regression in MIG6-knockout cells, small tumors remained and gradually regrew (Figure 8B). Panitumumab monotherapy could not induce tumor shrinkage. Combination therapy with crizotinib and panitumumab resulted in tumor shrinkage within 1 week, without severe weight loss (Supplemental Figure 18B and Supplemental Figure 19). Thus, these findings suggest that MIG6 depletion conferred resistance to ROS1-TKIs and that combination therapy with ROS1-TKIs and panitumumab was similarly effective in vivo.

## Discussion

This study conducted an unbiased genome-wide CRISPR library screening to identify genes that contribute to ALK-TKI resistance in ALK-rearranged lung cancer patient–derived cells, and multiple candidate genes were found. Among these candidate genes, we discovered that MIG6 loss induced drug persistence to ALK-TKI–naive cells through the activation of EGFR signaling. Similar to ALK-rearranged NSCLC, ROS1 fusion–positive NSCLC cell lines also showed resistance by MIG6 knockout. Furthermore, with MIG6 depletion, even low doses of EGFR ligands substantially enhanced cell survival and proliferation. Interestingly, MIG6 expression was downregulated in several ROS1-positive cell lines, namely, JFCR-168. Despite parental cell lines developing ROS1-TKI resistance in response to modest EGF doses, ROS1-TKI sensitivity was restored by MIG6 overexpression. In addition, in vivo and in vitro data demonstrated that combination therapy with the anti-EGFR antibody panitumumab induced substantial growth inhibition of resistant cells. Our results shed light on the resistance mechanism of ALK and ROS1-TKIs in NSCLC.

Excessive ErbB activity disrupts tissue homeostasis and leads to tumor proliferation, invasion, and metastasis. MIG6 directly binds to the C-lobe of the kinase domain of EGFR to inhibit EGFR activity, triggered by ErbB signaling (61, 62). The downregulation of MIG6 expression was reported to promote tumorigenesis and tumor invasion in various cancers, including lung cancer, breast cancer, and glioblastoma (63–66). However, evidence shows that high MIG6 levels are associated with resistance mechanisms in colorectal cancer and EGFR-mutated NSCLC (67, 68). Whether MIG6 expression affects the therapeutic response to molecular target therapy in fusion gene–positive NSCLC is still unknown. Our results support the role of MIG6 in the development of resistance to TKI, mainly due to the activation of the EGFR pathway. Consistent with a previous report (19), stimulation of HER3, as well as EGFR, might be essential, as MIG6 controls feedback in all members of the ErbB family. Indeed, completely restored sensitivity to ALK-TKIs was observed when resistant cells were treated with the combination therapy of ALK-TKIs and afatinib, a pan-ErbB inhibitor. Recent studies have demonstrated that MIG6 interacts with multiple cellular partners and mediates various biological processes in addition to controlling ErbB signaling. For example, MIG6 localized at the nuclei regulates DNA damage response in an ATM-dependent manner (69). Therefore, these MIG6 functions might be also associated with MIG6 depletion–related resistance mechanisms.

High levels of receptor tyrosine kinase ligands could induce resistance to TKIs, although the concentrations of ligands in these experiments (100 ng/mL) were much higher than the serum concentrations (54). The serum concentration of EGF has been reported to be approximately 150 pg/mL in healthy adults (70) and approximately 750–1,000 pg/mL in patients with advanced NSCLC (55, 71). EGFR ligands, such as EGF, TGF-α, and HB-EGF, are physiologically present in picomolar range (less than 1–2 ng/mL) in most human tissues and tumors (72, 73). Our data demonstrated that MIG6 depletion conferred substantial resistance to ALK-TKIs in the presence of EGFR ligands at physiological concentration. These results indicate that MIG6 plays a key role in preventing resistance to ALK-TKIs through the EGFR pathway. However, which cell types secrete EGFR ligands is still unknown. A possibility is autocrine related; in vitro data showed increased expression levels of HB-EGF (74) and TGF-α (22) in established ALK-TKI–resistant cells. The other possibility is paracrine related to the tumor microenvironment or systemic production; endothelial cells (75) and cancer-associated fibroblasts (76) secrete EGFR ligands. More studies including analysis of tumor microenvironments are warranted to elucidate the mechanism of EGFR ligand production.

Previous research indicated that varying MIG6 levels are expressed by different NSCLC cell lines; H322 lacks MIG6, whereas H23 expresses remarkably high MIG6 levels (77). Our in vitro data showed that MIG6 expression was relatively high in JFCR-028-3 and HCC78 cells, whereas it was relatively low in JFCR-168 cells. Therefore, even low doses of EGF induce resistance through the EGFR pathway in low MIG6–expressing cell lines. In these cell lines, MIG6 overexpression could also restore sensitivity to molecular target therapy. These results suggest that MIG6 might function as a barrier to prevent adaptive resistance through the EGFR pathway.

Many researchers have attempted to enhance the efficacy of molecular target therapy by inhibiting the EGFR signaling. In colorectal cancer, combined BRAF, EGFR, and MEK inhibition in patients resulted in modest improvement in response rates (78). Preclinical investigations have demonstrated the efficacy of combination therapy with ALK-TKIs and EGFR inhibitors, such as afatinib, for the adaptive resistance through the EGFR pathway (22), A recent study showed that combination therapy with ALK-TKIs and EGFR-TKIs might be more effective in the initial phase than in the TKI resistance phase (74). Consistently, our in vivo data indicated that combination therapy with ALK-TKIs and panitumumab inhibited the development of tumor recurrence following treatment termination. However, whether this combination is clinically effective and tolerable remains unclear. Small-scale studies have demonstrated that patients treated with EGFR-TKIs and cetuximab, an anti-EGFR antibody, experienced clinical improvement and tolerable adverse events (79, 80). More studies are necessary to evaluate this combination in the clinical setting.

Slow cell cycling and proliferative activity and reversibility of drug sensitivity are 2 common phenotypic characteristics of DTP. These features convincingly imply that nongenomic or epigenetic processes might be responsible for the acquisition of the DTP phenotype. Receptor tyrosine kinases were reported to mediate the DTP state through epigenetic modification. For example, EGFR-TKI therapy induces the activation of histone demethylase and transcriptomic factor, which leads to the upregulation of IGF-1R, resulting in a DTP state (21, 40). Cancer cell survival has also been linked to chromosomal instability triggered by the overexpression of AURKA (81), a crucial regulator of cellular mitosis, and FGFR3 (82), which promotes EMT programming. In BRAF-mutant melanoma, the dynamics of ERK signaling and DTP formation are directly influenced by the kinetics of receptor tyrosine kinase activation (83). MIG6 could be regulated through epigenetic or transcriptomic mechanisms, leading to acquired resistance; however, its precise mechanism remains unclear. Given that DTP cells are in a relatively dormant state, functional loss of MIG6 might be a key factor for the DTP to resume proliferative signaling. The most pertinent analogy to the experimental DTP state is the occurrence of minimal residual disease in patients with advanced cancer receiving molecular target therapies. Despite a favorable initial response to therapy, these responses are generally partial and are followed by a protracted period, during which the remaining tumor lesions on radiological imaging appear dormant. Since obtaining tissue samples of patients with minimal residual disease is challenging, we lack an understanding of the nature and the role of DTP in clinical settings. Thus, more studies are needed to establish treatment strategies targeting DTP.

This study has several limitations. First, there is little evidence that MIG6 depletion is correlated with clinical outcomes. Second, how DTP cells acquire dependency on EGFR signaling remains unclear. Third, which cell is responsible for the secretion of EGFR ligands in the in vivo model was not identified.

In summary, we identified loss of MIG6 as a resistance mechanism to ALK- and ROS1-TKIs using CRISPR/Cas9 library screening.

## Methods

### Cell lines and culture condition.

Human embryonic kidney 293FT cells (Thermo Fisher Scientific) were cultured in high-glucose DMEM (Fujifilm Wako) supplemented with 10% fetal bovine serum (FBS). H3122 cells, which were gifted by JA Engelman (Massachusetts General Hospital Cancer Center, Boston, Massachusetts, USA), were cultured in RPMI-1640 medium (Wako Pure Chemical Industries) supplemented with 10% FBS and 100 μg/mL of kanamycin. HCC78 was obtained from DSMZ (Germany). HCC78xe3 ROS1-WT cell is a subclone of HCC78, generated by repeating subcutaneous implantation and in vitro cell culture 3 times, and induced SLC34A2-ROS1 overexpression as previously described (84). ALK fusion–positive and ROS1 fusion–positive NSCLC PDC lines were established from the patients’ pleural effusion. All patients provided informed consent for the genetic and cell biological analyses, which were performed in accordance with a protocol approved by the Institutional Review Board of the JFCR. NSCLC PDC lines JFCR-028-3 and JFCR-168 and HCC78 were cultured in RPMI and Ham’s F12 medium with 10 mM HEPES (Nacalai Tesque), 15% FBS, and 1× antibiotic-antimycotic mixed stock solution (Nacalai Tesque).

### Reagents.

Lorlatinib, crizotinib, gilteritinib, and brigatinib were purchased from Shanghai Biochempartner Co., Ltd. Alectinib was purchased from ActiveBiochem. Taletrectinib and entrectinib were synthesized at DaiichiSankyo Co., Ltd. Afatinib was obtained from ChemieTek. Entrectinib was purchased from MedChemExpress. PP242 was bought from AdooQ Bioscience. Osimertinib was purchased from Selleck. Panitumumab was procured from Takeda Pharm. The human recombinants EGF, HB-EGF, and TGF-α were purchased from PeproTech. Brigatinib was dissolved in ethanol, and the other inhibitors were dissolved in dimethyl sulfoxide (DMSO) for the cell culture experiments.

### Cell viability assay.

To evaluate cell viability, cells were seeded in triplicate at a density of 3,000 cells/well in 96-well plates. JFCR-168 cells were seeded in 96-well collagen-coated plates (IWAKI), and HCC78 cells were in ultralow-attachment dishes (3262, Corning). Cells were treated with panitumumab for 10 hours and stimulated with the indicated concentrations of EGFR ligands. After 72 hours of drug treatment, the cells were incubated with CellTiter-Glo assay reagent (Promega) for 10 minutes. Luminescence was measured using a Tristar LB 941 microplate luminometer (Berthold Technologies) or Centro LB960 microplate luminometer (Berthold Technologies). GraphPad Prism version 7.04 (GraphPad Software) was used to analyze and graphically display the data.

### Caspase activity assay.

Cells were seeded in triplicate at a density of 3,000 cells/well in 96-well, collagen-coated plates (IWAKI). Following 48 hours of drug treatment, cells were incubated with Caspase-Glo assay reagent (Promega) for 60 minutes. Luminescence was measured using a Tristar LB 941 microplate luminometer (Berthold Technologies). GraphPad Prism version 7.04 (GraphPad Software) was used to analyze and graphically display the data.

### Western blotting and antibodies.

Western blotting was performed as previously described (85). Cells were seeded at a density of 5 × 10^5^ cells/well in 6-well plates, 6-well collagen-coated plates (IWAKI), or 6-well ultralow-attachment dishes (Corning) and treated with the indicated drug concentrations. Lysates were prepared using 1× sodium dodecylsulfate (SDS) lysis buffer (1% SDS and 10% glycerol in 100 mM Tris-HCl [pH 7.5]) or RIPA buffer (50 mM Tris-HCl [pH 7.4], 150 mM sodium chloride, 0.5% sodium deoxycholate, 0.1% SDS, 1% NP-40 substitute, 1 mM EDTA, and 10 mM sodium fluoride) with protease and phosphatase inhibitors (Roche). Protein quantification of cell lysates was performed using a bicinchoninic acid protein assay kit (Thermo Fisher Scientific) according to the manufacturer’s instructions, and luminescence was measured using a Multiskan GO Microplate Spectrophotometer (Thermo Fisher Scientific). The cell lysates were adjusted to equal amounts of proteins using an SDS lysis buffer, and a 20% volume of 5× sample buffer containing 0.65 M Tris-HCl (pH 6.8), 20% 2-mercaptoethanol, 10% glycerol, 3% SDS, and 0.01% bromophenol blue was added. Equal amounts of proteins were added to SDS-PAGE and then immunoblotted. The following antibodies were purchased from Cell Signaling Technology: total EGFR (4627, 1:1,000), total ALK (3633, 1:2,000), phospho-ALK (Y1604, 3341, 1:1,000. Y1278, 6941, 1:1,000), total ROS1 (69D6, 3266, 1:2,000), phospho-ROS1 (Y2274, 3078, 1:1,000), total AKT (4691, 1:1,000), phospho-AKT (S473, 4060, 1:1,000), total p42/44 ERK/MAPK (9102, 1:1,000), phospho-p42/44 ERK/MAPK (T202/Y204, 9101, 1:2,000), total S6 ribosomal protein (2217, 1:1,000), phospho-S6 ribosomal protein (S240/244, 5364, 1:8,000), NF2 (6695, 1:1,000), and MED12 (14360, 1:1,000). In addition, GAPDH antibody was purchased from MilliporeSigma (MAB374, 1:5,000), total MIG6 antibody was purchased from Proteintech (11630-1-AP, 1:1,000), and phospho-EGFR antibody was purchased from GeneTex (132810, 1:1,000). ECL Prime Western Blotting Detection Reagent (GE Healthcare, now Cytiva) or SuperSignal West Femto Maximum Sensitivity Substrate (Thermo Fisher Scientific) was used for signal detection. The signals were detected using Amersham Imager 600 (GE Healthcare) or Amersham Imager 800 (GE Healthcare).

### Colony formation assays.

Colony formation assays were conducted of 2 × 10^3^, 1 × 10^4^, 2 × 10^4^, and 1 × 10^5^ cells per well of HCC78, H3122, JFCR-028-3, and JFCR-168, respectively, into 12-well plates. After 48 hours of seeding, cells were treated with the indicated inhibitors. The medium was changed every 2–3 days, and cells were cultured with inhibitors for 9 days to 2 weeks. Colonies were fixed in 4% paraformaldehyde phosphate-buffered solution (Wako) for 15 minutes at room temperature and stained with 0.5% crystal violet (Sigma) for 30 minutes at room temperature. After staining, pictures of the wells were taken. The crystal violet dye was solubilized in 30% ethanol and 1% acetic acid, then measured by absorbance at 570 nm using a Multiskan GO Microplate Spectrophotometer.

### RT-qPCR.

RNA was extracted from the cells using the RNeasy Mini Kit (QIAGEN), and cDNA was synthesized from the extracted RNA using ReverTra Ace qPCR RT Master Mix (Toyobo) according to the manufacturer’s protocol. The synthesized cDNA was used for the template and mixed with FastStart Essential DNA Green Master kit (Roche) and target-specific primers, and sequences are shown in Supplemental Table 2. RT-qPCR was performed using LightCycler 96 (Roche). GAPDH was used for control and the relative expression level of each gene was calculated as the 2-ΔΔCt.

### Flow cytometry analysis.

A total of 5 × 10^5^ cells were prepared in 100 μL FACS buffer (PBS with 0.5% BSA). Then 1 μL of PE-Cy7–conjugated anti-EpCAM antibody (324222, BioLegend) or isotype control–PE-Cy7 (M8894, MilliporeSigma) was added and incubated for 30 minutes on ice. Measurement was performed using FACSMelody (BD Biosciences), and data were analyzed using FlowJo software (TMOY Digital Biology).

### Genome-wide CRISPR/Cas9 knockout library screen.

First, a stable Cas9-expressing JFCR-028-3 cell line was established by the lentiviral transduction of the Cas9-coding sequence. Following a 1-week selection in the presence of 7 μg/mL of blasticidin, we performed single-cell cloning. Knockout efficacy was evaluated using sgRNA targeting EpCAM. Cas9 expression was confirmed by Western blotting. Second, cells were transduced with the human GeCKO v2 library that contains 58,029 unique sgRNA sequences targeting 19,052 human genes (3 sgRNAs per gene and 1,000 nontargeting controls) at a low MOI (~0.1) to ensure effective barcoding of each cell. Then, the transduced cells were selected with 2 μg/mL of puromycin for 7 days to generate a mutant cell pool, which was then treated with vehicle (DMSO), 300 nmol/L of alectinib, or 100 nmol/L of lorlatinib for 9 days, respectively. After treatment, at least 1 × 10^7^ cells were collected for genomic DNA extraction to ensure over 5× coverage of the GeCKO v2 library. The sgRNA sequences were amplified using KOD-plus-NEO (Toyobo) and the following primers: LentiGuide_sgRNA_ilR1: TCGTCGGCAGCGTCAGATGTGTATAAGAGACAGCTTGTGGAAAGGACGAAACAC, LentiGuide_sgRNA_ilR2: GTCTCGTGGGCTCGGAGATGTGTATAAGAGACAGTTCAAGTTGATAACGGACTAGCC. Each library was subjected to tagmentation using Nextera XT Index Kit v2 set A (Illumina). The samples were subjected to massive parallel amplicon sequencing conducted by HiSeq X Ten (Illumina). The sgRNA read counts and hit calling were analyzed by MAGeCK ver 5.4 algorithm.

### Establishment of MIG6-knockout cell lines.

Stable Cas9-expressing JFCR-028-3 and H3122 cell lines were established by the lentiviral transduction of the Cas9-coding sequence. Cas9 expression was confirmed by a Cas9-specific antibody (7A9-3A3, 1:1,000, Cell Signaling Technology). The MIG6- and NF2-knockout cell lines were established by overexpressing sgRNA targeting the coding sequence of each gene. Plasmids carrying Cas9 and sgRNA were lentiCas9-Blast (52962, Addgene) and lentiGuide-Puro (52963, Addgene). For the establishment of MIG6 knockout in HCC78 and JFCR-168 cells, lentiCRISPRv2-Puro (98290, Addgene) was used. Viruses were replicated in 293FT cells by transfecting with packaging plasmids. After 24 hours of viral transduction, the cells were selected by incubation with 1.5 μg/mL (JFCR-028-3 and H3122) or 1 μg/mL (JFCR-168 and HCC78) of puromycin. sgRNA sequences are shown in Supplemental Table 3.

### Generating lentivirus and stable MIG6 expression in JFCR-168 cells.

cDNA encoding MIG6 was amplified by PCR and cloned into a pENTR (Thermo Fisher Scientific) vector, then cloned into pLenti6.3 (Thermo Fisher Scientific) using LR clonase II. Lipofectamine 2000 (Thermo Fisher Scientific) was used to make lentivirus by transfecting pLenti6.3 construct in 293FT cells, following the manufacturer’s protocol. JFCR-168 cells were selected by incubation with 10 μg/mL of blasticidin for 1 week. pLenti6.3/V5-DEST-EGFP (Thermo Fisher Scientific) was used for control.

### Microarray analysis.

Total RNA was extracted from the resected lung tumor samples using RNeasy Mini Kit (QIAGEN) according to manufacturer’s protocol. RNAs were applied to the slides and analyzed on the Agilent 028004 SurePrint G3 Human GE 8x60K. Background correction and quantile normalization were conducted using RStudio (Posit).

### Animals and subcutaneous xenograft model.

All animal studies were performed in line with animal protocols approved by the Institutional Animal Care and Use Committee and institutional guidelines. Specific pathogen–free 5-week female BALB/c nude mice were purchased from Charles River Laboratories Japan, Inc. (Yokohama, Japan). In vitro–cultured cells (2.5 × 10^6^) were transplanted subcutaneously into the mouse dorsum. HCC78xeno3 SLC34A2-ROS1-WT cells were suspended in HBSS containing a 50% matrix growth factor reduced. After the tumor volume reached approximately 200 mm^3^, crizotinib (50 mg/kg) or alectinib (10 mg/kg) was orally administered 5 days per week for 4 weeks. Panitumumab (0.5 mg/mice) was administered via intraperitoneal injections twice a week for 4 weeks. The tumor volume was calculated as length × width^2^ × 0.5 (mm^3^).

### Statistics.

Data were analyzed using GraphPad Prism version 7.04. In vitro data are presented as the mean ± SD. Xenograft tumor progression was expressed as the mean ± SEM. Statistical significance among > 3 groups was determined using the 1-way ANOVA or 2-way ANOVA followed by Dunnett’s multiple-comparison test. Two-sided *P* values less than 0.05 were considered significant.

### Study approval.

All patients provided informed consent for the genetic and cell biological analyses, which were performed in accordance with protocol approved by the Institutional Review Board of the JFCR. All in vivo studies were conducted according to protocols approved by the Committee for the Use and Care of Experimental Animals of the JFCR.

### Data availability.

Transcriptomic data obtained from the microarray analysis of patients’ samples have been deposited in National Center for Biotechnology Information Gene Expression Omnibus under the accession code GSE128309. Deep sequencing data of sgRNA presented in this work will be submitted to the Sequencing Read Archive. All the other data supporting the findings of this study are available within the article and its Supporting Data Values file and from the corresponding author upon reasonable request.

## Author contributions

RK designed the study, performed cell line experiments, supervised the experiments, and wrote the manuscript. NK designed the study; performed cell line, in vitro, and in vivo experiments; and wrote the manuscript. TU designed the study and performed next-generation sequencing analysis. YS and TO performed cell line experiments. KU and MN identified the patients, obtained patients’ specimens, and established PDC lines. SSM, HN, and KT collected patients’ samples and performed microarray analysis. AT performed in vivo experiments. YM supervised the experiments and edited the manuscript.

## Supplementary Material

Supplemental data

Supporting data values

## Version 1 11/02/2023

In-Press Preview

## Version 2 12/22/2023

Electronic publication
