# Supplementary material for: MIG6 loss confers resistance to ALK/ROS1 inhibitors in NSCLC through EGFR activation by low-dose EGF
Source: JCI Insight. 2023 Dec 22;8(24):e173688. doi: 10.1172/jci.insight.173688 (PMC10807714; doi:10.1172/jci.insight.173688)
Supplement: Supplemental data [file jciinsight-8-173688-s032.pdf]

Supplementary Table 1

Clinical features of biopsy samples used for RT-qPCR

Patient ID	Biopsy ID	Diagnosis	Previous ALK-TKI	Resistance	Biopsy sample	Putative resistance mechanism
JFCR-028	028-3	LUAD	Crizotinib	Pre-alectinib	Pleural effusion	
	028-5		Alectinib	Alectinib-resistance	Pleural effusion	Unknown (Activation of Src)
JFCR-134	134-1	LUAD	Crizotinib	Crizotinib-resistance	EBUS-TBNA	
	134-5		Lorlatinib	Lorlatinib-resistance	Left lower lung	G1202R+L1196M
JFCR-248	248-1	LUAD	No	Treatment naïve	Pleural effusion	
	248-4		Alectinib	Alectinib-resistance	Pleural effusion	Unknown
JFCR-276	276-2	LUAD	Alectinib	Alectinib-resistance	EBUS-TBNA	
	276-6		Lorlatinib	Lorlatinib-resistance	Lymph node	ALK I1171N
JFCR-426	426-2	LUAD	No	Treatment naïve	Pleural effusion	
	426-8		Alectinib	Alectinib-resistance	Pleural effusion	Unknown

LUAD; lung adenocarcinoma

EBUS-TBNA; endobronchial ultrasound-guided transbronchial needle aspiration

Supplementary Table 2

The information of the primers using for RT-qPCR

Primer name	sequence
EGF_Forward	TGCGATGCCAAGCAGTCTGTGA
EGF_Reverse	GCATAGCCCAATCTGAGAACCAC
TGFA_Forward	GGTCCGAAAACACTGTGAGTGG
TGFA_Reverse	CAAACTCCTCCTCTGGGCTCTT
GAPDH_Forward	TCAGCCGCATCTTCTTTTGC
GAPDH_Reverse	TTAAAAGCAGCCCTGGTGAC
ERRFI1_Forward	GCGAAGGATCTGCCAGTAAG
ERRFI1_Reverse	AGGTATGGTGGTCGTTCAGG

Supplementary Table 3

The information of sgRNA using for knockout cell lines

sgRNA	Sequence
sgMIG6-1	CTCGGTGTGCGCGAGTTACT
sgMIG6-3	AGGTTCTCTTGGCGGTACTT
sgNF2	CCTGGCTTCTTACGCCGTCC
sgCtrl	ACGGAGGCTAAGCGTCGCAA
sgEpCAM	GTGCACCAACTGAAGTACAC

Fig.S1

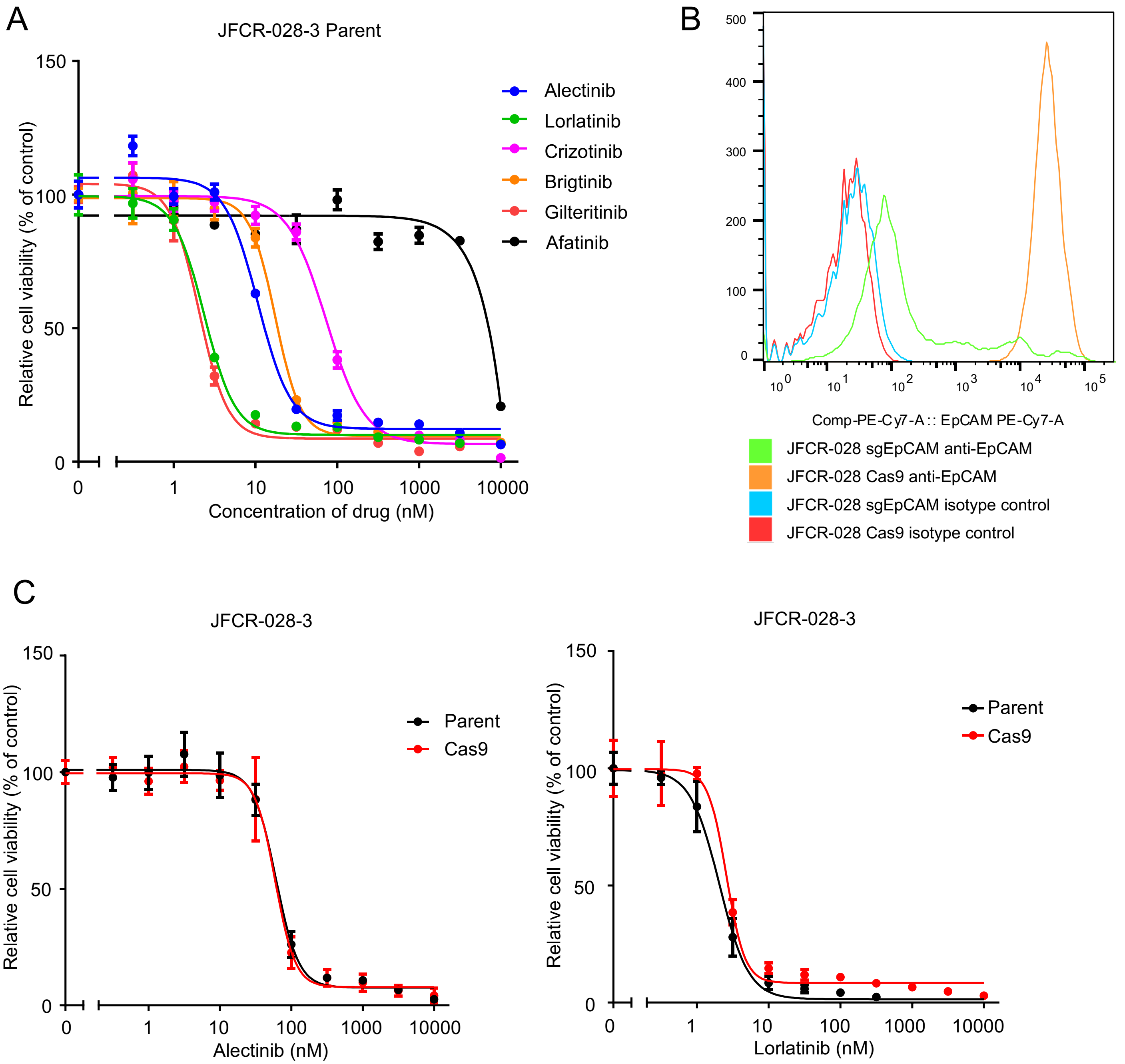

Supplementary Figure 1
Characteristic of JFCR-028-3 cells
(A), JFCR-028-3 parental cells were treated with the indicated concentrations of ALK-TKIs and afatinib for 72 h. Cell viability was measured using the CellTiter-Glo assay. Each point represents the mean ± SD of three technical replicates. **(B)**, Flow cytometry was performed to confirm the knockout efficacy of EpCAM. **(C)**, JFCR-028-3 parental and Cas9 stable-expressing cells were treated with the indicated concentrations of ALK-TKIs for 72 h. Cell viability was measured using the CellTiter-Glo assay. Each point represents the mean ± SD of three replicates. **(A-C)**, Similar experiments were performed twice and representative data are shown.

Fig.S2

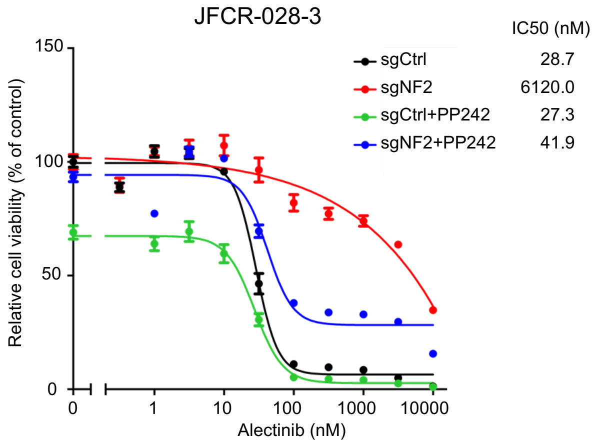

Supplementary Figure 2
NF2 depletion confers resistance to alectinib
JFCR-028-3 cells were treated with the indicated concentrations of alectinib for 72 h. Cell viability was measured using the CellTiter-Glo assay (n = 3). Each point represents the mean ± SD of three technical replicates., Similar experiments were performed 3 times and representative data are shown.

Fig.S3

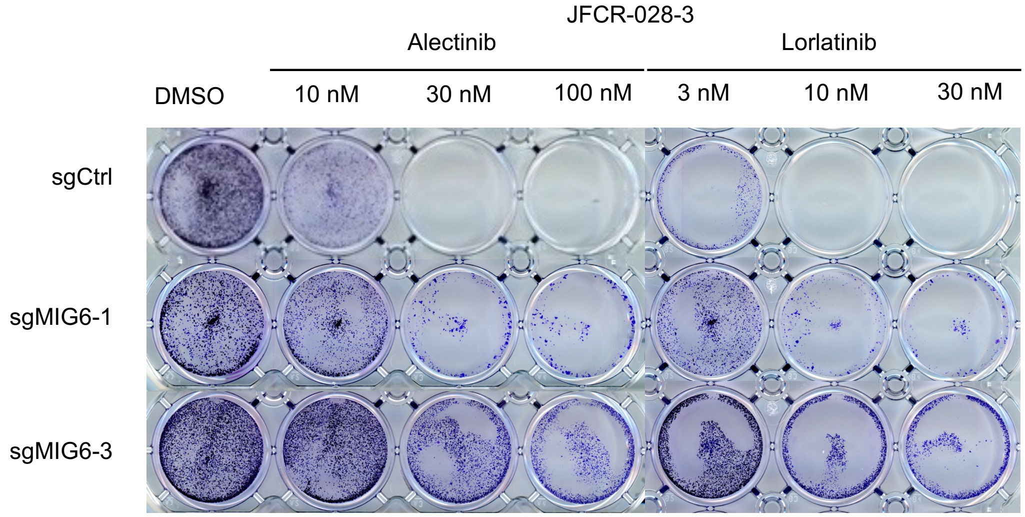

Supplementary Figure 3
MIG6 depletion confers resistance to ALK-TKIs
Colony formation assays were performed in JFCR-028-3 cells. JFCR-028-3 sg-control or sg-MIG6 cells were treated with various concentrations of alectinib or lorlatinib for 2 weeks using 3 technical replicates. Surviving cells were stained with crystal violet. Representative images are shown., Similar experiments were performed twice and representative data are shown.

Fig.S4

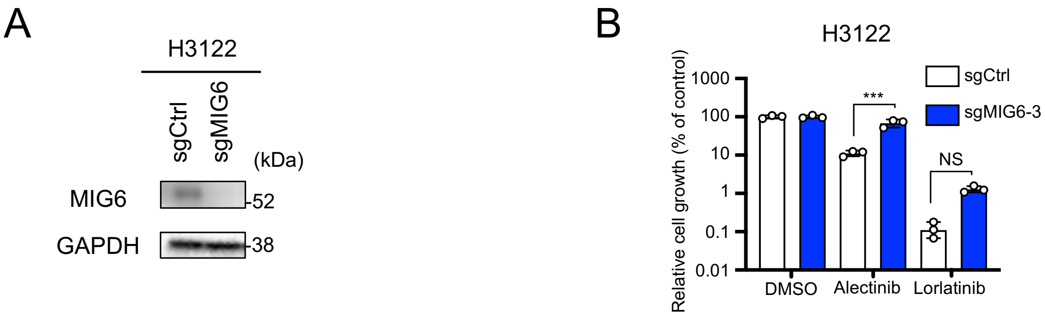

Supplementary Figure 4
MIG6 depletion in H3122 conferred resistance to ALK-TKIs
(A), immunoblot analysis of MIG6 knocked out in H3122 cells. **(B)**, Colony formation assays were performed in H3122 cells. Each well was treated with 30 nmol/L of alectinib or 30 nmol/L of lorlatinib for 2 weeks and surviving cells were stained with crystal violet. Relative cell viability was measured using a spectrophotometer after solubilizing the stained crystal violet with an acetic acid buffer from each well. The results indicate the mean ± SD of three technical replicates; ***$p < 0.001$ (two-way ANOVA following Dunnett's post-hoc test). **(A and B)**, Similar experiments were performed twice, and representative data are shown.

Fig.S5

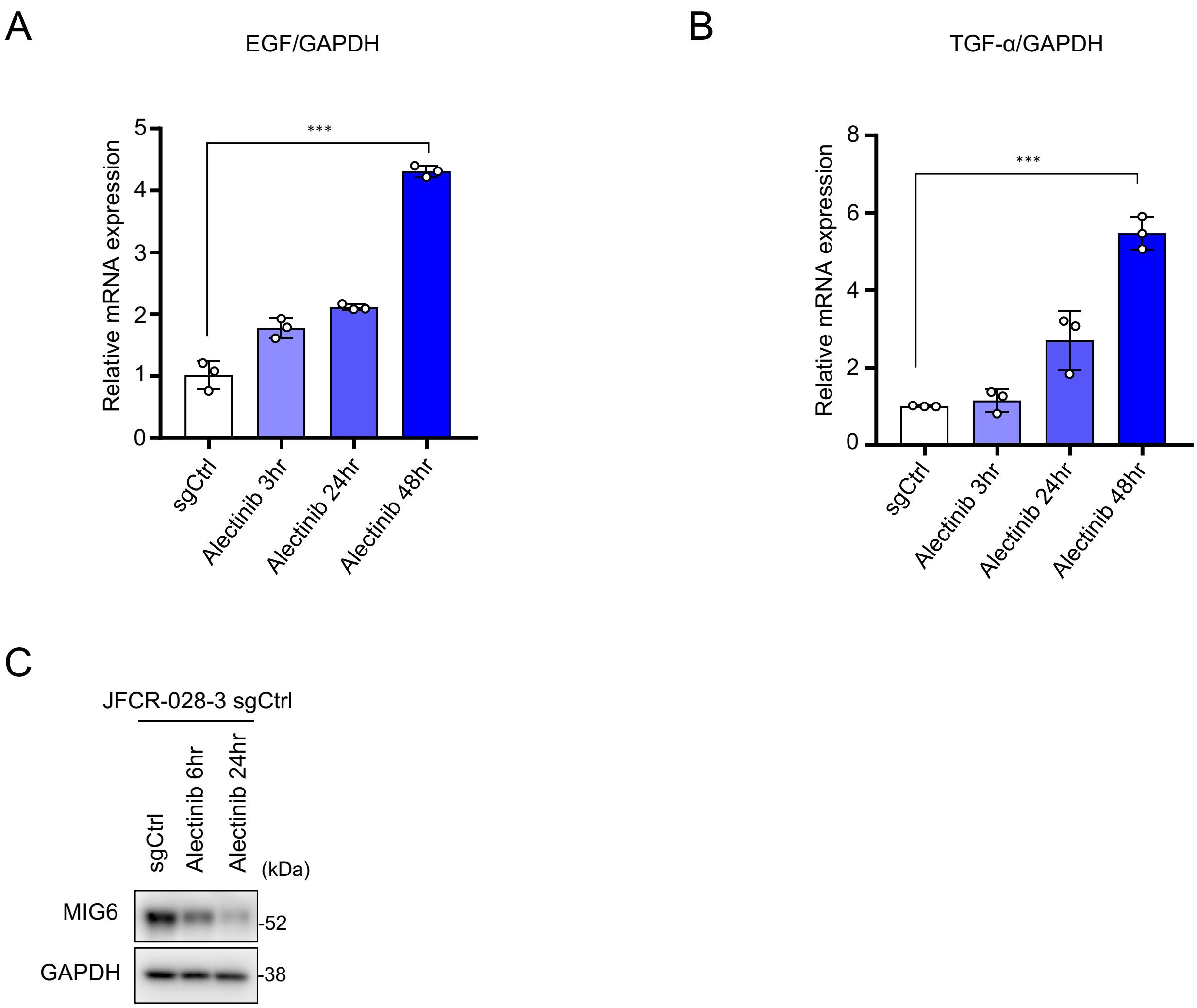

Supplementary Figure 5
ALK-TKI therapy induced the increased levels of EGFR ligands
(A and B), Quantitative RT-PCR of EGF and TGFA RNA was performed using JFCR-028-3 cells treated with 300 nmol/L of alectinib for the indicated hours. Each point represents the relative mRNA expression of MIG6/GAPDH shown as mean ± SD of three technical replicates; ***$p < 0.001$ (two-way ANOVA following Dunnett's post-hoc test). **(C)**, immunoblot analysis of MIG6 treated with 100 nmol/L of alectinib for the indicated hours., Similar experiments were performed 3 times and representative data are shown.

Fig.S6

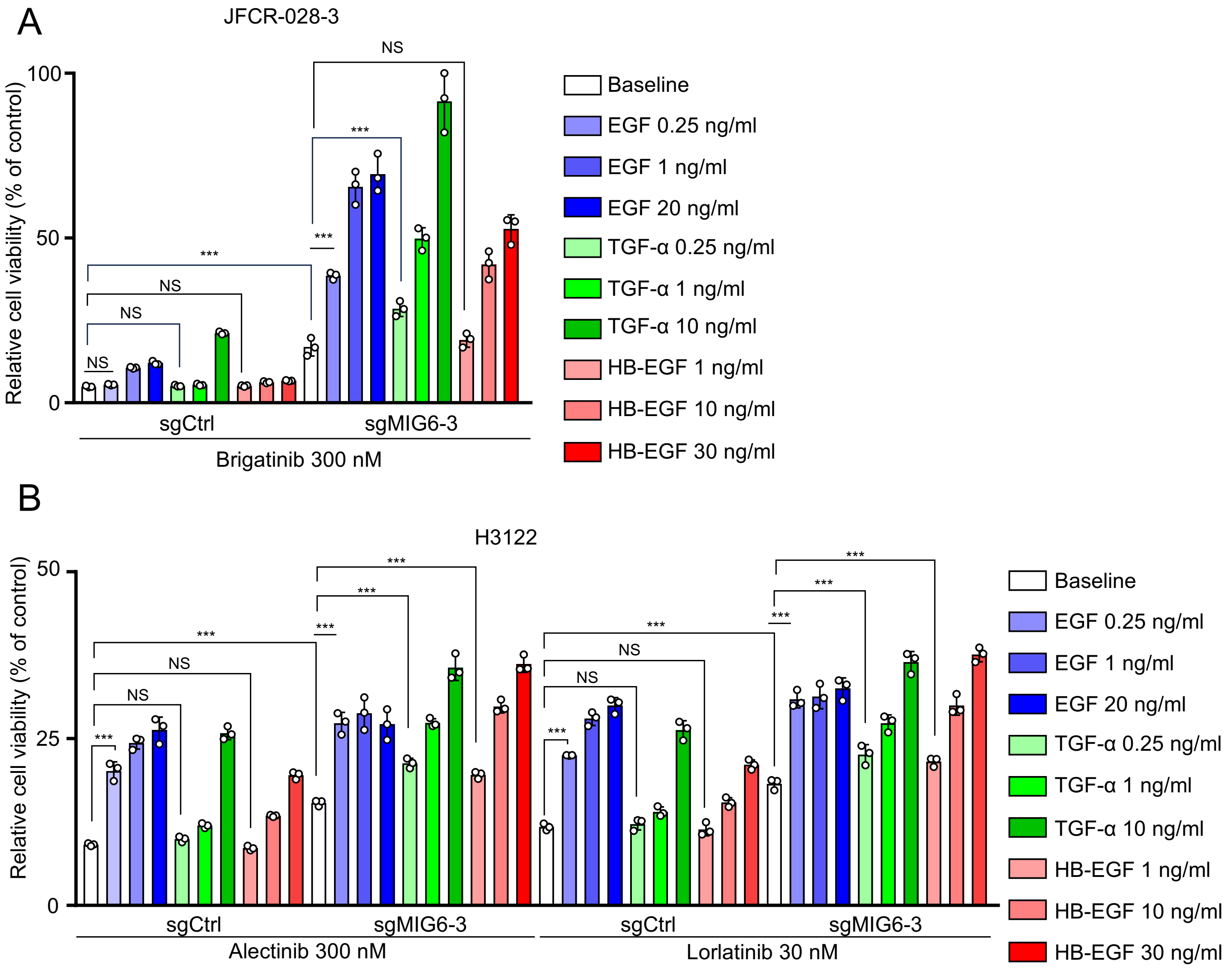

Supplementary Figure 6

Low-dose EGFR ligands confer more resistance to various ALK-TKIs in MIG6-knockout cells

JFCR-028-3 (A) or H3122 (B) cells were treated with the indicated concentrations of drugs and ligands for 72 h. Cell viability was measured using the CellTiter-Glo assay. Each point represents the mean ± SD of three technical replicates; ***$p < 0.001$ (two-way ANOVA following Tukey's post-hoc test). Similar experiments were performed twice and representative data are shown.

Fig.S7

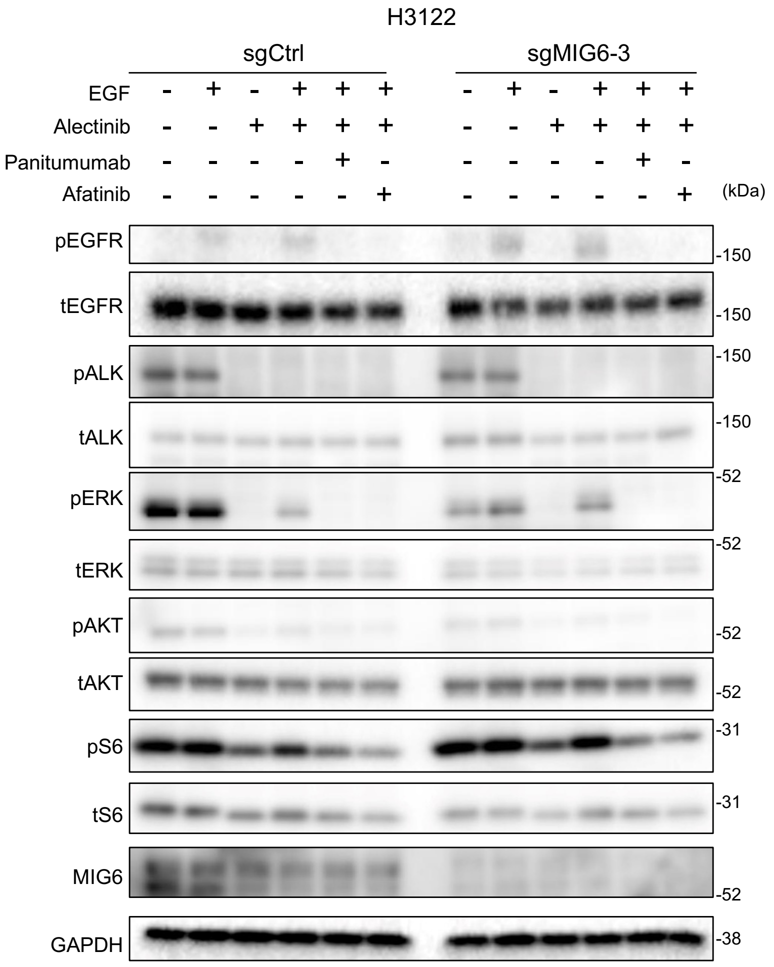

Supplementary Figure 7

Combination of EGF and ALK-TKIs induced the activation of the downstream pathway of ALK in MIG6-knockout H3122 cells

Western blotting analysis of H3122 cells treated with 300 nmol/L of alectinib, 10 µg/mL of panitumumab, 100 nmol/L of afatinib and 1 ng/mL of EGF for 3 h. Similar experiments were performed twice and representative data are shown.

Fig.S8

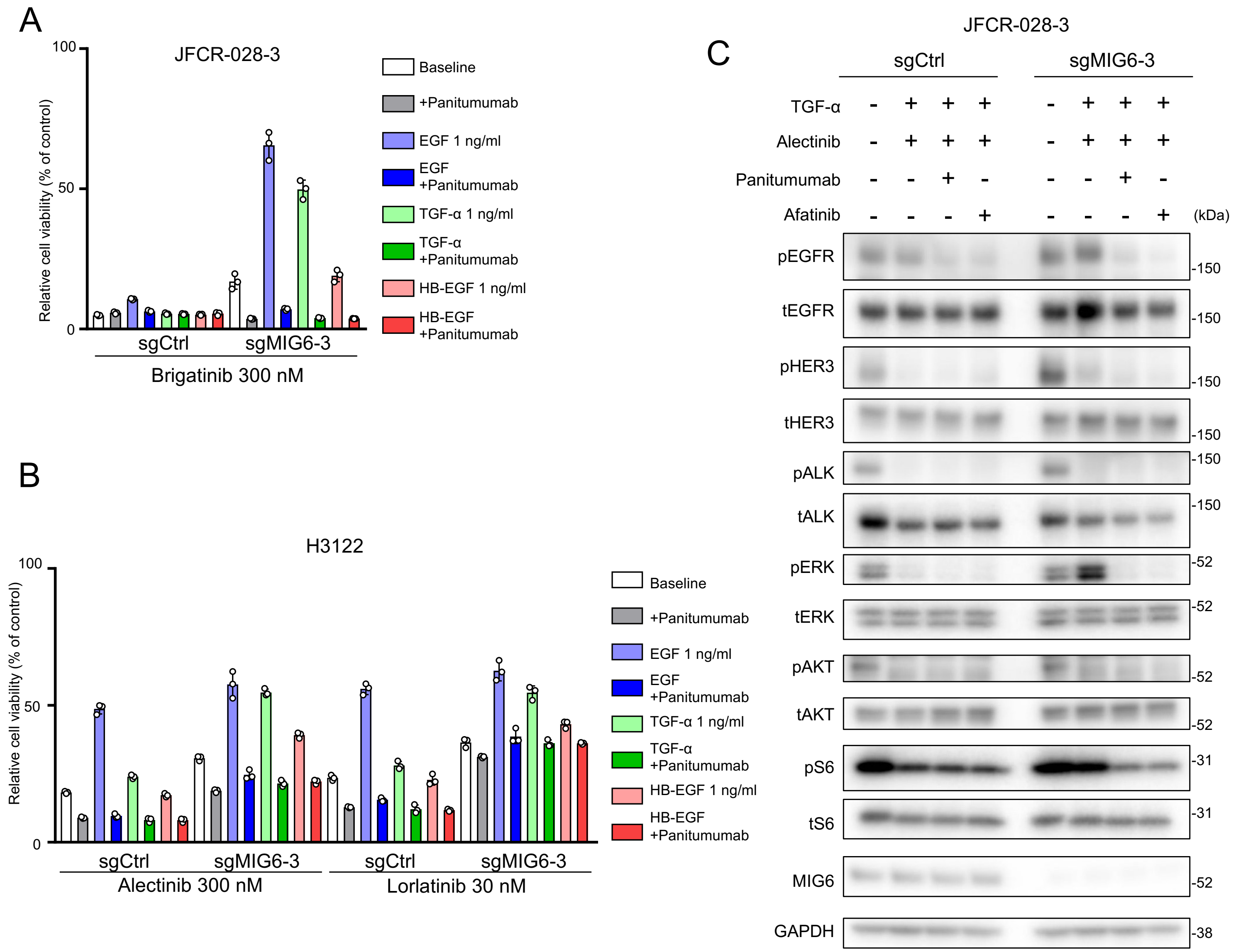

Supplementary Figure 8

Combination therapy with EGFR inhibitors and various ALK-TKIs could overcome MIG6 depletion-related resistance

(A and B), JFCR-028-3 (A) and H3122 (B) cells were treated with the indicated concentrations of ALK-TKIs and ligands with or without 10 μg/mL of panitumumab for 72 h. Cell viability was measured using the CellTiter-Glo assay. Each point represents the mean ± SD of three technical replicates. **(C)**, Protein expression of the downstream pathway of ALK in JCFR-028-3 cells. Cells were treated with 300 nmol/L of alectinib, 10 μg/mL of panitumumab, 100 nmol/L of afatinib and 1 ng/mL of TGF-α for 3 h. **(A-C)**, Similar experiments were performed twice (A and B) or 3 times (C), and representative data are shown.

Fig.S9

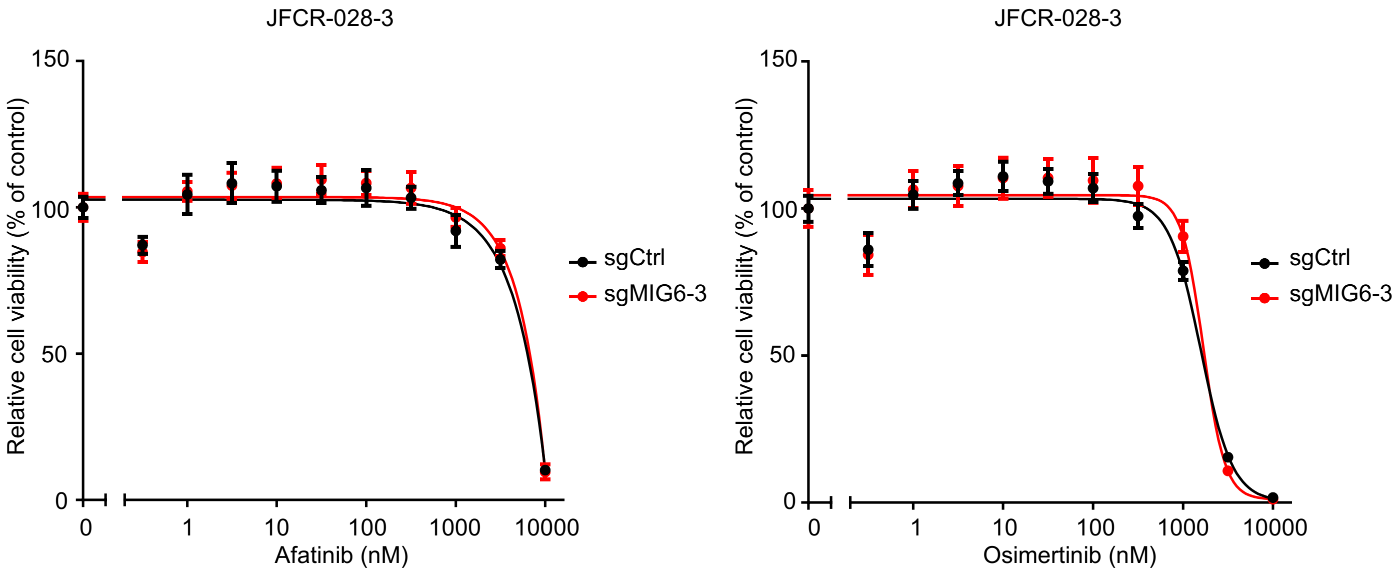

Supplementary Figure 9
JFCR-028-3 MIG6-knockout cells were resistant to EGFR-TKIs.
JFCR-028-3 cells were treated with the indicated concentrations of drugs and ligands for 72 h. Cell viability was measured using the CellTiter-Glo assay. Each point represents the mean ± SD of three technical replicates. Similar experiments were performed twice and representative data are shown.

Fig.S10

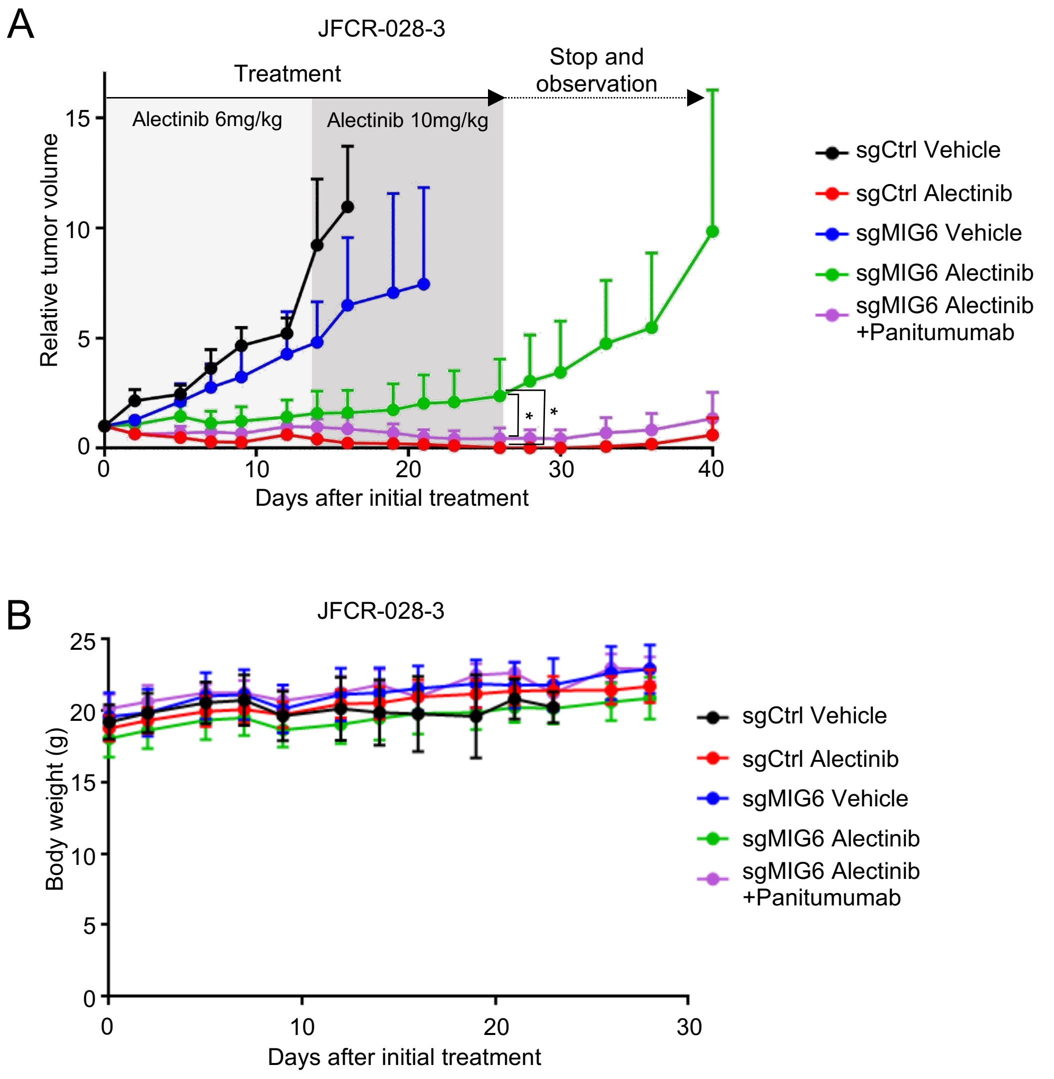

Supplementary Figure 10
MIG6 depletion conferred resistance to ALK inhibitors in vivo
(A), JFCR-028-3 control and MIG6 knockout cells were subcutaneously injected into BALB/c nude mice. The mice were treated with vehicle, alectinib (first 2week; 6 mg/kg, following 10 mg/kg) orally or alectinib plus panitumumab (0.5 mg, twice a week) intraperitoneally for 4 weeks (n = 5). Data are presented as the mean ± SEM; * $p < 0.05$ (one-way ANOVA following Dunnett's test). **(B)**, Body weight was measured daily.

Fig.S11

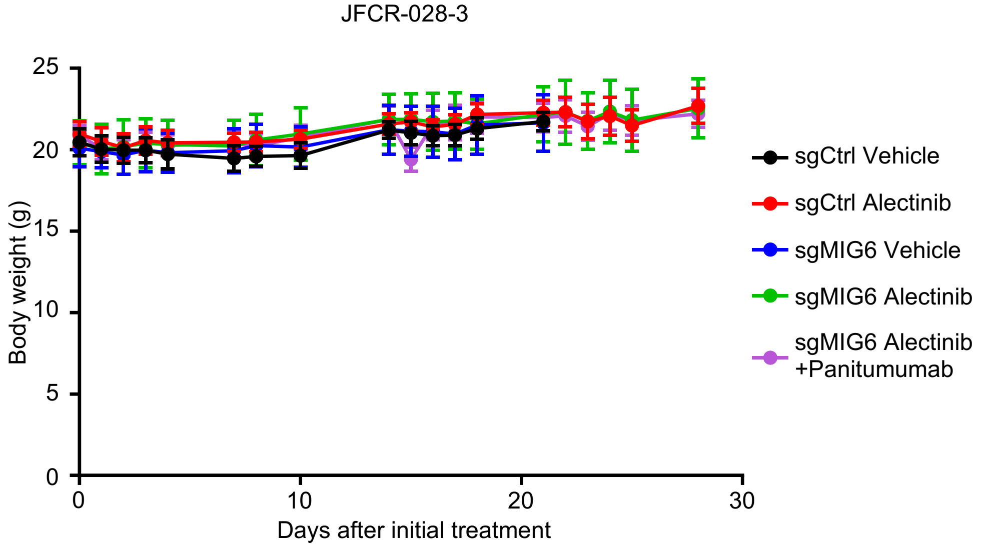

Supplementary Figure 11

Combination therapy with alectinib and panitumumab did not induce severe weight loss

JFCR-028-3 cells were subcutaneously injected into nude mice and the mice were treated with vehicle, alectinib (10 mg/kg) orally or alectinib plus panitumumab (0.5 mg, twice a week) intraperitoneally for 4 weeks (n = 6). Body weight was measured daily.

Fig.S12

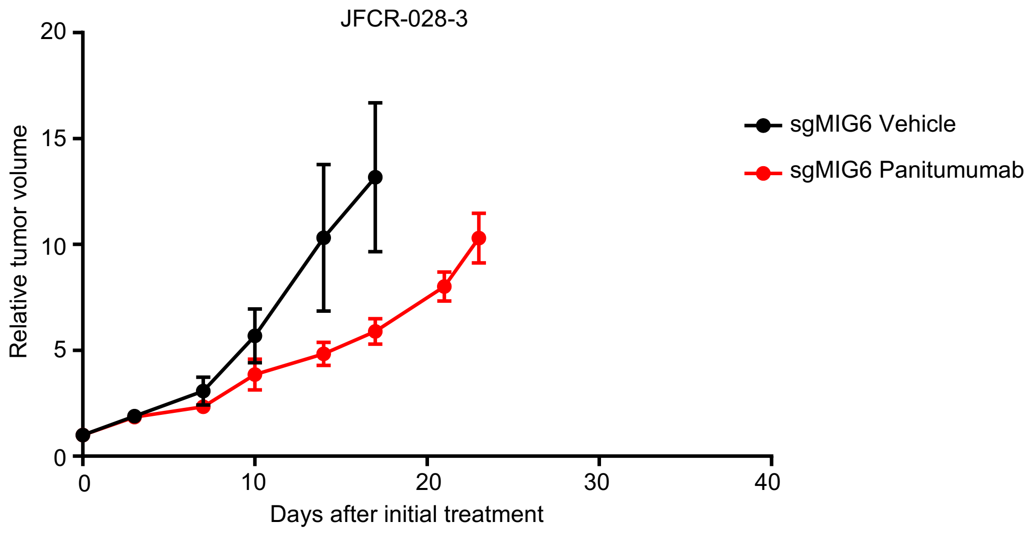

Supplementary Figure 12

Panitumumab monotherapy failed to overcome the MIG6 depletion-related resistance in vivo

JFCR-028-3 MIG6-knockout cells were subcutaneously injected into BALB/c nude mice. The mice were treated with vehicle or panitumumab (0.5 mg, twice a week) intraperitoneally for 3 weeks ($n = 4$). Data are presented as the mean ± SEM.

Fig.S13

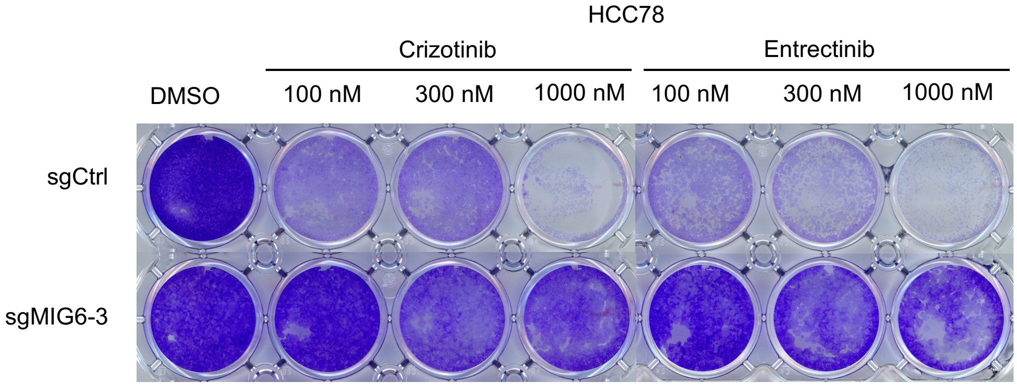

Supplementary Figure 13
MIG6 depletion confers resistance to ROS1-TKIs
Colony formation assays were performed in HCC78 cells. HCC78 sg-control or sg-MIG6 cells were treated with various concentrations of crizotinib or entrectinib for 2 weeks using 3 technical replicates. Surviving cells were stained with crystal violet. Representative images are shown., Similar experiments were performed twice and representative data are shown.

Fig.S14

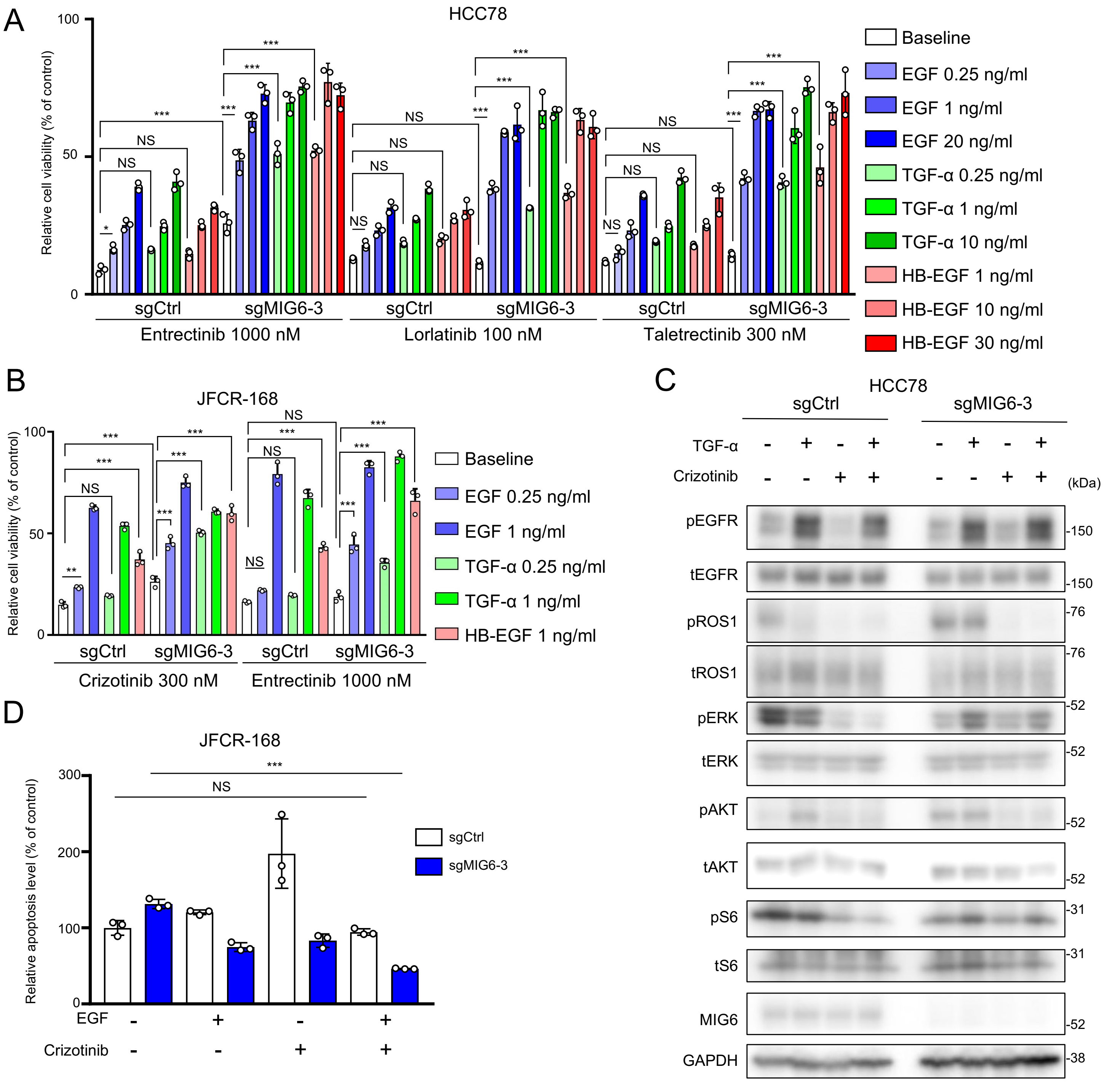

Supplementary Figure 14

Low-dose EGFR ligands confer more resistance to various ROS1-TKIs in MIG6-knockout cells

(A and B), HCC78 (A) or JFCR-168 (B) cells were treated with the indicated concentrations of drugs and ligands for 72 h. Cell viability was measured using the CellTiter-Glo assay. **(C)**, Protein expression of the downstream pathway of ROS1 in HCC78 cells. Cells were treated with 1000 nmol/L of crizotinib for 3 h and 20 ng/mL of TGF-α for the indicated hours. **(D)**, JFCR-168 cells were treated with 1000 nmol/L of crizotinib and 1 ng/mL of EGF for 48 h. Apoptosis level was measured using the Caspase-Glo assay. **(A-D)**, Each point represents the mean ± SD of three technical replicates; ***$p < 0.001$ (two-way ANOVA following Tukey's post-hoc test). Similar experiments were performed twice and representative data are shown.

Fig.S15

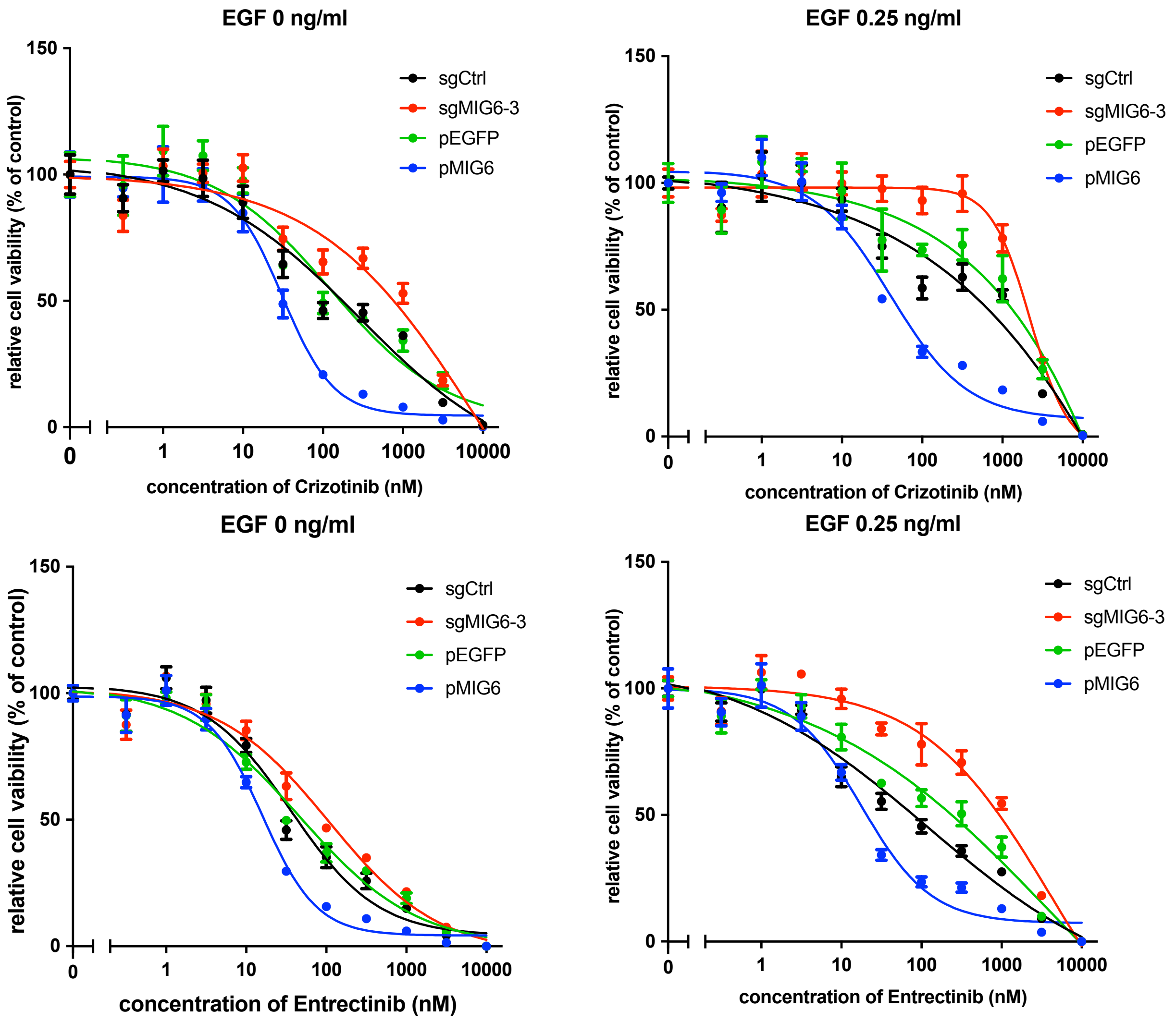

Supplementary Figure 15

JFCR-168 cells could restore the sensitivity to ROS1-TKIs by MIG6 overexpression

JFCR-168 cells were treated with the indicated concentrations of drugs and ligands for 72 h. Cell viability was measured using the CellTiter-Glo assay. Each point represents the mean ± SD of three technical replicates. Similar experiments were performed twice and representative data are shown.

Fig.S16

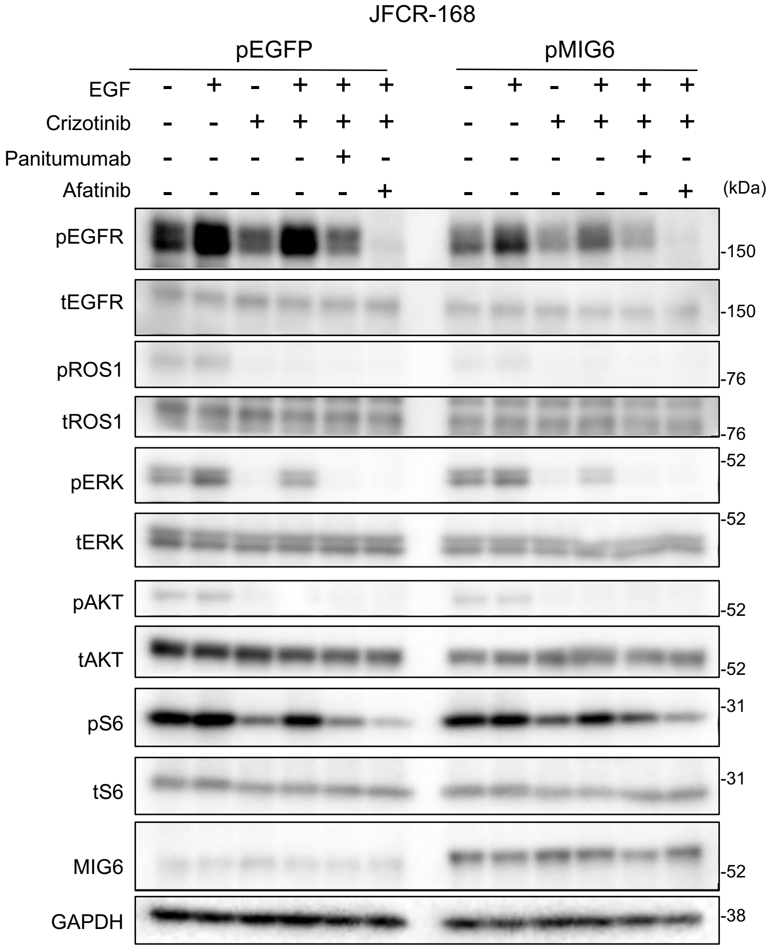

Supplementary Figure 16
Combination of EGF and ROS1-TKIs suppressed the activation of the downstream pathway of ROS1 in MIG6-overexpressed JFCR-168 cells
Western blotting analysis of JFCR-168 cells treated with 1000 nmol/L of crizotinib, 10 µg/mL of panitumumab, 100 nmol/L of afatinib and 1 ng/mL of EGF for 3 h., Similar experiments were performed twice and representative data are shown.

Fig.S17

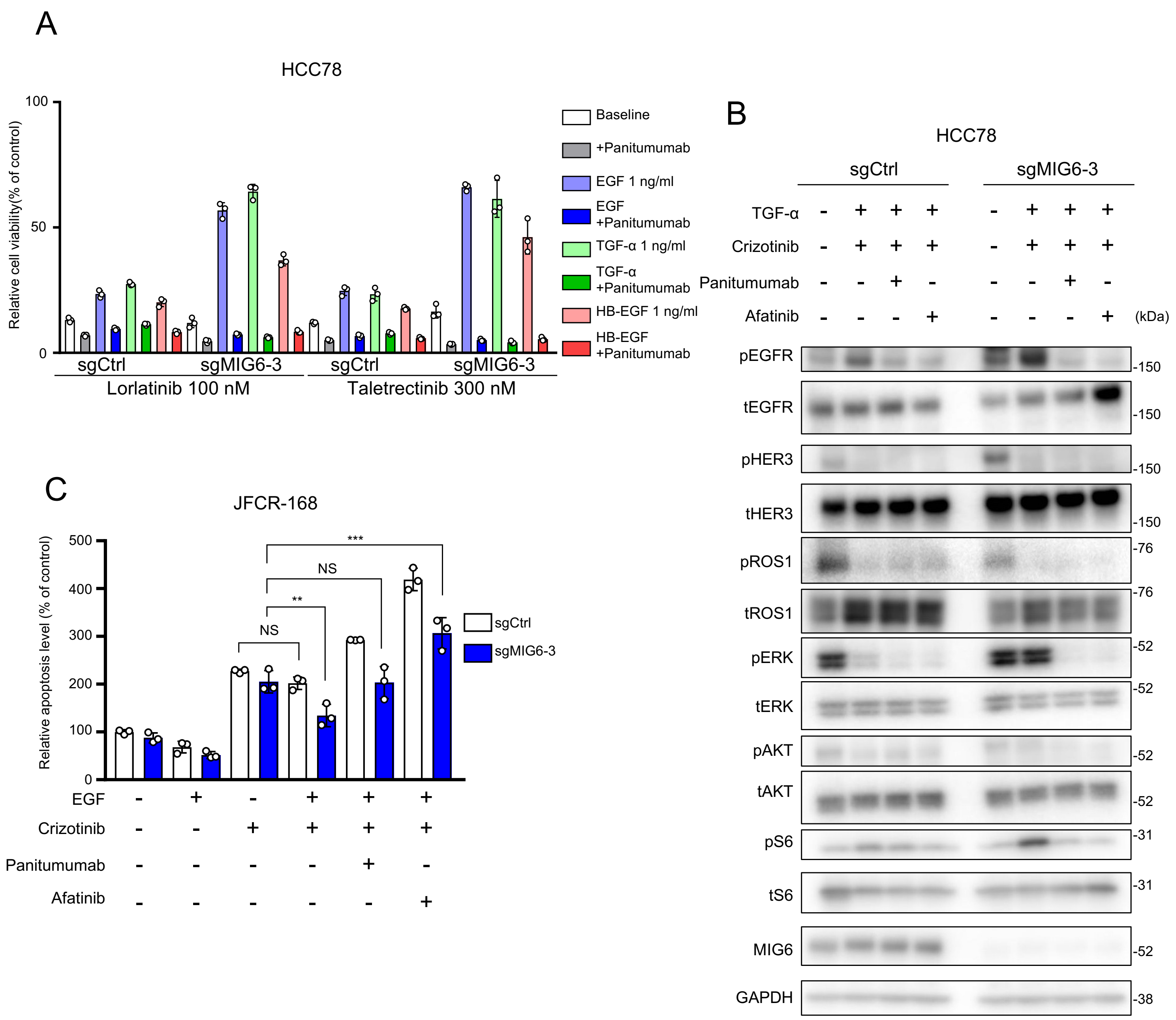

Supplementary Figure 17

Combination therapy with EGFR inhibitors and various ROS1-TKIs could overcome MIG6 depletion-related resistance

(A), HCC78 cells were treated with the indicated concentrations of ROS1-TKI and ligands with or without 10 μg/mL of panitumumab for 72 h. Cell viability was measured using the CellTiter-Glo assay. **(B)**, Protein expression of the downstream pathway of ROS1 in HCC78 cells. Cells were treated with 1000 nmol/L of crizotinib, 10 μg/mL of panitumumab, 100 nmol/L of afatinib and 1 ng/mL of TGF-α for 3 h. **(C)**, JFCR-168 cells were treated with 1000 nmol/L of crizotinib, 10 μg/mL of panitumumab, 100 nmol/L of afatinib and 1 ng/mL of EGF for 48 h. Apoptosis level was measured using the Caspase-Glo assay. **(A-C)**, Each point represents the mean ± SD of three technical replicates; $^{**}p < 0.01$, $^{***}p < 0.001$ (two-way ANOVA following Tukey's post-hoc test). Similar experiments were performed twice and representative data are shown.

Fig.S18

A

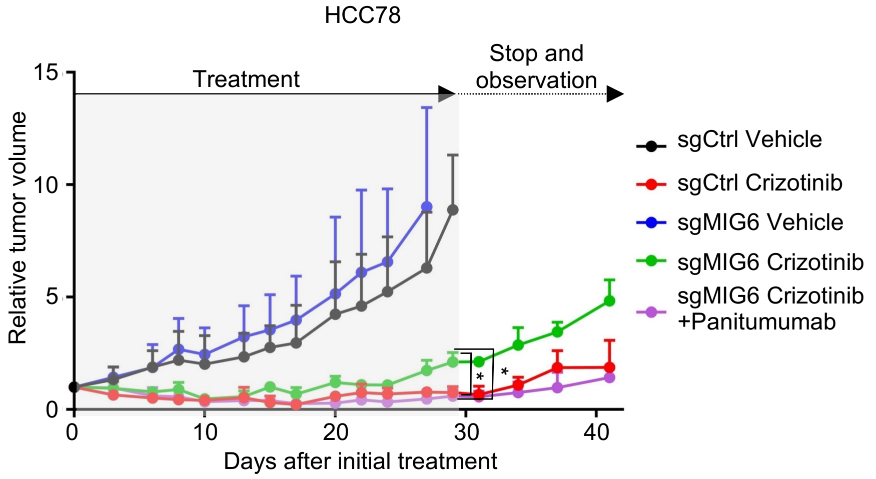

B

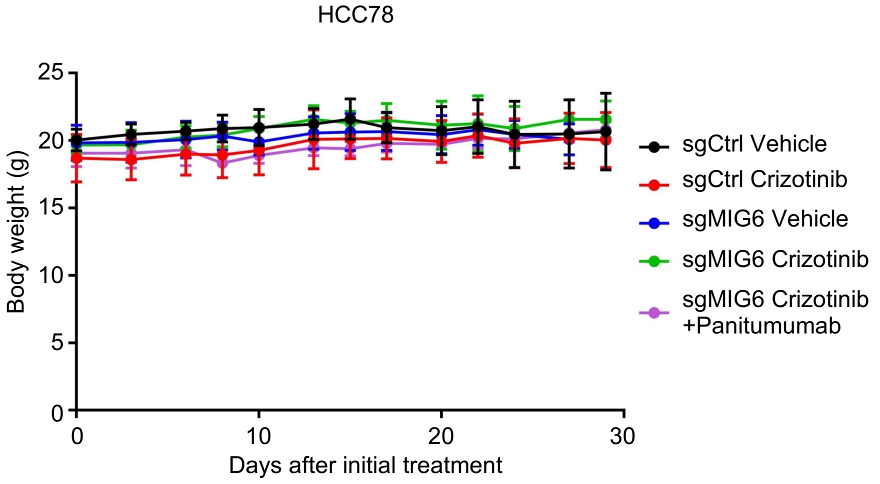

Supplementary Figure 18

Antitumor effect of ROS1-TKIs in the HCC78 xenograft MIG6-depletion model

(A), HCC78 control and MIG6-knockout cells were subcutaneously transplanted into BALB/c nude mice. The mice were treated with vehicle, crizotinib (50 mg/kg) orally or crizotinib plus panitumumab (0.5 mg, twice a week) intraperitoneally for 4 weeks (n = 3). Data are presented as mean ± SEM; *p < 0.05 (one-way ANOVA following Dunnett's test). **(B)**, Body weight was measured every day.

Fig.S19

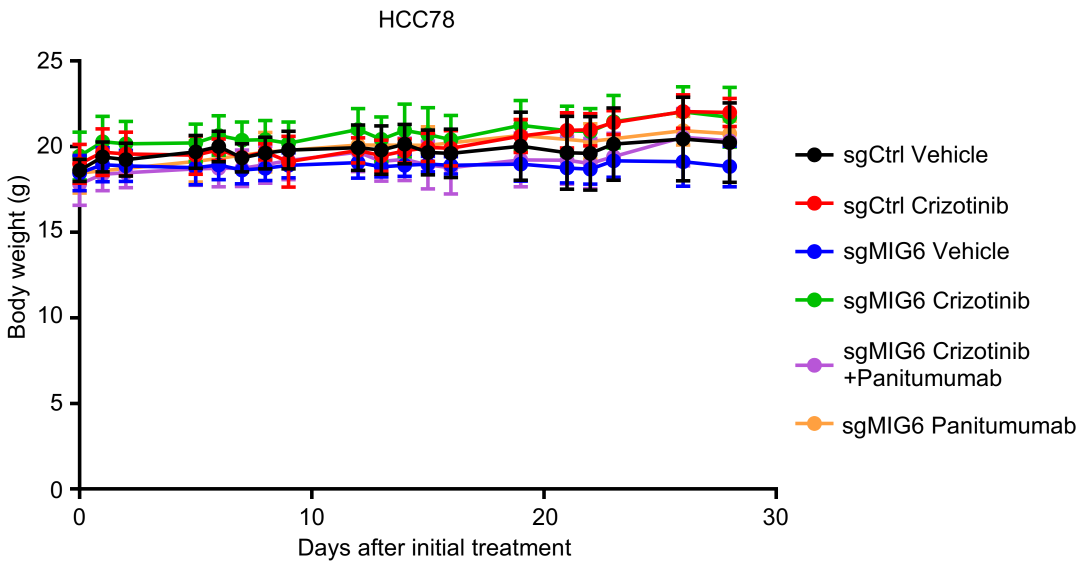

Supplementary Figure 19
Combination therapy with crizotinib and panitumumab did not induce severe weight loss
HCC78 cells were subcutaneously injected into nude mice and the mice was treated with vehicle, crizotinib (50 mg/kg) orally, panitumumab (0.5 mg, twice a week) intraperitoneally or crizotinib plus panitumumab for 4 weeks (n = 5–6). Body weight was measured daily.
